# Combination Therapy of Curcumin and Disulfiram Synergistically Inhibits the Growth of B16-F10 Melanoma Cells by Inducing Oxidative Stress

**DOI:** 10.3390/biom12111600

**Published:** 2022-10-31

**Authors:** Sheila S. Fontes, Mateus L. Nogueira, Rosane B. Dias, Clarissa A. Gurgel Rocha, Milena B. P. Soares, Marcos A. Vannier-Santos, Daniel P. Bezerra

**Affiliations:** 1 Gonçalo Moniz Institute, Oswaldo Cruz Foundation (IGM-FIOCRUZ/BA), Salvador 40296-710, BA, Brazil; 2 Department of Propedeutics, School of Dentistry of the Federal University of Bahia, Salvador 40110-909, BA, Brazil; 3 SENAI Institute for Innovation in Advanced Health Systems, SENAI CIMATEC, Salvador 41650-010, BA, Brazil; 4 Oswaldo Cruz Institute, LITEB, Oswaldo Cruz Foundation, Rio de Janeiro 21040-360, RJ, Brazil

**Keywords:** curcumin, disulfiram, melanoma, apoptosis, oxidative stress

## Abstract

Oxidative stress plays a central role in the pathophysiology of melanoma. Curcumin (CUR) is a polyphenolic phytochemical that stimulates reactive oxygen species (ROS) production, while disulfiram (DSS) is a US FDA-approved drug for the treatment of alcoholism that can act by inhibiting the intracellular antioxidant system. Therefore, we hypothesized that they act synergistically against melanoma cells. Herein, we aimed to study the antitumor potential of the combination of CUR with DSS in B16-F10 melanoma cells using in vitro and in vivo models. The cytotoxic effects of different combination ratios of CUR and DSS were evaluated using the Alamar Blue method, allowing the production of isobolograms. Apoptosis detection, DNA fragmentation, cell cycle distribution, and mitochondrial superoxide levels were quantified by flow cytometry. Tumor development in vivo was evaluated using C57BL/6 mice bearing B16-F10 cells. The combinations ratios of 1:2, 1:3, and 2:3 showed synergic effects. B16-F10 cells treated with these combinations showed improved apoptotic cell death and DNA fragmentation. Enhanced mitochondrial superoxide levels were observed at combination ratios of 1:2 and 1:3, indicating increased oxidative stress. In vivo tumor growth inhibition for CUR (20 mg/kg), DSS (60 mg/kg), and their combination were 17.0%, 19.8%, and 28.8%, respectively. This study provided data on the potential cytotoxic activity of the combination of CUR with DSS and may provide a useful tool for the development of a therapeutic combination against melanoma.

## 1. Introduction

Melanoma is one of the most aggressive forms of malignant skin neoplasms and one of the main causes of cancer mortality. Although it represents only 4% of dermatological cancers, it is responsible for 80% of skin cancer deaths due to its high metastatic capacity and high refractoriness to chemotherapy [1,2,3,4]. The 5-year survival rate of patients with metastatic melanoma is less than 20% [5]. Dacarbazine, an alkylating agent, is the main treatment for advanced melanoma. However, serious side effects have been observed, and the therapeutic response rate is approximately 10% [5,6]. These results indicate that new therapies for melanoma are urgently required.

Oxidative stress plays a central role in the pathophysiology of melanoma since the generation of melanin leads to the generation of hydrogen peroxide and consumption of reduced glutathione (GSH) [7,8]. Consequently, melanoma maintains high baseline ROS levels compared to normal cells, which makes melanoma cells more susceptible to oxidative stress [7,9].

Curcumin (CUR) is a polyphenolic phytochemical isolated from turmeric, a food spice made from the rhizome of *Curcuma longa* L. [10]. Turmeric is traditionally used in many South Asian countries, both in traditional medicine and cooking, and has been used for over 2000 years as a medicine in China and India [11]. Curiously, many studies have reported that CUR causes cell death by stimulating reactive oxygen species (ROS) production in fibroblasts [12], leukemia [13], lymphoma [14], melanoma [15], and colon cancer [16].

Disulfiram (DSS) is a thiocarbamate drug approved by the United States Food and Drug Administration (US-FDA) for the clinical treatment of alcoholism and has been used for over 60 years [17]. DSS is an irreversible inhibitor of aldehyde dehydrogenase (ALDH). Interestingly, ALDH can decrease intracellular oxidative stress due to its ROS scavenger action. Consequently, DSS can act by inhibiting the intracellular antioxidant system [18,19].

Importantly, both DSS and CUR not only have antitumor activities but can also potentiate the action of anticancer chemotherapy drugs by blocking drug efflux pumps [20,21,22,23,24,25,26]. Furthermore, these compounds have been reported as agents capable of reducing the adverse reactions of highly toxic chemotherapeutic agents [27,28].

Therefore, we hypothesized that CUR, an oxidative stress inducer [29], combined with DSS, which acts as an antagonist of the intracellular antioxidant system [22], will have a synergistic effect, providing greater sensitization of cancer cells and resulting in cell death. Herein, we aimed to study the antitumor potential of the combination of CUR with DSS in B16-F10 melanoma cells using in vitro and in vivo models.

## 2. Material and Methods

### 2.1. CUR and DSS Obtaining

CUR and DSS were purchased from Sigma-Aldrich (Sigma-Aldrich Co., Saint Louis, MO, USA).

### 2.2. In Vitro Assays

#### 2.2.1. Cells

The mouse melanoma B16-F10 cell line and human lung fibroblast MRC-5 cell line were obtained from the American Type Culture Collection (ATCC, Manassas, VA, USA) and were cultured as recommended by the ATCC animal cell culture guide. All cell lines were tested for mycoplasma using a mycoplasma staining kit (Sigma-Aldrich Co.) and were free from contamination. Cell viability was examined using the trypan blue exclusion method for all experiments. Over 90% of the cells were viable at the beginning of culture.

#### 2.2.2. Alamar Blue Method

The quantification of cell viability was carried out by the Alamar Blue method, as previously described in refs. [30,31,32]. Briefly, the cells were seeded in 96-well plates and incubated for 72 h. CUR and DSS were tested on a concentration-response curve obtained by serial dilution of a stock dissolved in dimethyl sulfoxide (DMSO; Vetec Química Fina Ltd.a, Duque de Caxias, RJ, Brazil), with concentrations based on previous studies [33,34,35]. Doxorubicin (DOX, doxorubicin hydrochloride, purity ≥ 95%, Laboratory IMA S.A.I.C., Buenos Aires, Argentina) was used as a positive control. At the end of the treatment, 20 µL of a stock solution (0.312 mg/mL) of resazurin (Sigma-Aldrich Co.) were added to each well. Absorbances at 570 and 600 nm were measured using a SpectraMax 190 Microplate Reader (Molecular Devices, Sunnyvale, CA, USA).

#### 2.2.3. Flow Cytometry Assays

To quantify cell death, the FITC Annexin V Apoptosis Detection Kit I (BD Biosciences, San Jose, CA, USA) was used, and the analysis was performed according to the manufacturer’s instructions. Cell fluorescence was determined by flow cytometry.

DNA fragmentation and cell cycle distribution were determined using 2 µg/mL propidium iodide (PI) in cells permeabilized with 0.1% Triton X-100, 0.1% sodium citrate and 100 µg/mL RNAse (all from Sigma-Aldrich Co.), as previously described in ref. [36], and cell fluorescence was assessed by flow cytometry.

Detection of superoxide levels was performed using MitoSOX™ Red reagent, a fluorogenic dye specifically targeted to mitochondria in living cells (Thermo Fisher Scientific, Waltham, MA, USA), and the analysis was performed according to the manufacturer’s instructions. Cell fluorescence was determined by flow cytometry.

For all analyses using flow cytometry, 10,000 events were recorded per sample with a BD LSRFortessa cytometer and analyzed with BD FACSDiva Software (BD Biosciences, San Jose, CA, USA) and FlowJo Software 10 (Flowjo LCC, Ashland, OR, USA), and cellular debris was omitted from the analysis.

### 2.3. In Vivo Assay

#### 2.3.1. Animals

A total of 50 specific pathogen-free C57BL/6 mice (males, 25–30 g) were obtained and maintained at the animal facilities of the Gonçalo Moniz Institute-FIOCRUZ (Salvador, BA, Brazil). Animals were housed in cages with free access to food and water. All animals were kept under a 12:12 h light–dark cycle (lights on at 6:00 a.m.). The animals were treated according to the ethical principles for animal experimentation of SBCAL (Brazilian Association of Laboratory Animal Science), Brazil. The Animal Ethics Committee of the Oswaldo Cruz Foundation (Salvador, BA, Brazil) approved the experimental protocol (number 01/2013).

#### 2.3.2. In Vivo B16-F10 Melanoma Model

The in vivo antitumor effect was evaluated in C57BL/6 mice inoculated with B16-F10 melanoma cells, as previously described in ref. [37]. Tumor cells (2 × 10^6^ cells per 500 μL) were implanted subcutaneously into the left hind groin of mice. The compounds were dissolved in 5% DMSO and provided to the mice intraperitoneally once a day for 15 consecutive days. Mice were divided into five groups at the beginning of the experiment: Group 1 (negative control, *n* = 10): animals treated with vehicle (5% DMSO); Group 2 (positive control, *n* = 10): animals treated with DOX (1 mg/kg/day); Group 3: animals treated with CUR (20 mg/kg/day, *n* = 10); Group 4: animals treated with DSS (60 mg/kg/day, *n* = 10); Group 5: animals treated with CUR (20 mg/kg/day) plus DSS (60 mg/kg/day) (*n* = 10). The treatments were initiated one day after tumor injection. On day 16, the animals were anesthetized (thiopental, 50 mg/kg), and samples of peripheral blood were collected from the brachial artery for hematological analyses, as described below. The animals were euthanized by anesthetic overdose (thiopental, 100 mg/kg), and the tumors were excised and weighed. Drug effects are expressed as the percentage inhibition in relation to the control.

#### 2.3.3. Systemic Toxicological Evaluation

Systemic toxicological effects were also investigated, as previously described in ref. [37]. The mice were weighed at the beginning and end of the experiment. The animals were observed for signs of abnormalities throughout the study. Hematological analyses were performed using an Advia 60 hematology system (Bayer, Leverkusen, Germany). Their livers, kidneys, lungs, and hearts were removed, weighed, and examined for any signs of gross lesions or color changes and hemorrhage. Following macroscopic analysis, representative tissue sections of the tumors, livers, kidneys, lungs, and hearts were fixed in 4% buffered formalin and embedded in paraffin. Tissue sections with a thickness of 4 µm were stained with hematoxylin and eosin, and the analyses were performed under light microscopy.

### 2.4. Statistical Analysis

Inhibitory concentrations of 50% (IC_50_) values and their 95% confidence intervals (CI 95%) were obtained via nonlinear regression. The fractional inhibitory concentration (FIC) was calculated following the formula FIC(a) = effect (a) of the compound in combination/effect (a) of the compound alone where (a) are the effects of 25%, 50%, and 75% inhibition resulting in FIC25, FIC50, and FIC75. Isobolograms were constructed using the coordinates formed by the FIC (CUR + DSS) of the 25%, 50%, and 75% effects. The line linking the number 1 in both axes was used. Points below this line indicate synergistic results of combination, and points above the line indicate antagonism. Points upon the line indicate an addictive effect [38].

Data are presented as the means ± SEMs or IC_50_ values. Differences between experimental groups were compared using ANOVA (analysis of variance) followed by the Student Newman-Keuls test (*p* < 0.05). Statistical analyses were performed using GraphPad software (GraphPad Software, Inc., San Diego, CA, USA).

## 3. Results

### 3.1. Combination Therapy of CUR and DSS Synergistically Inhibits the Growth of B16-F10 Melanoma Cells

**Figure 1:**
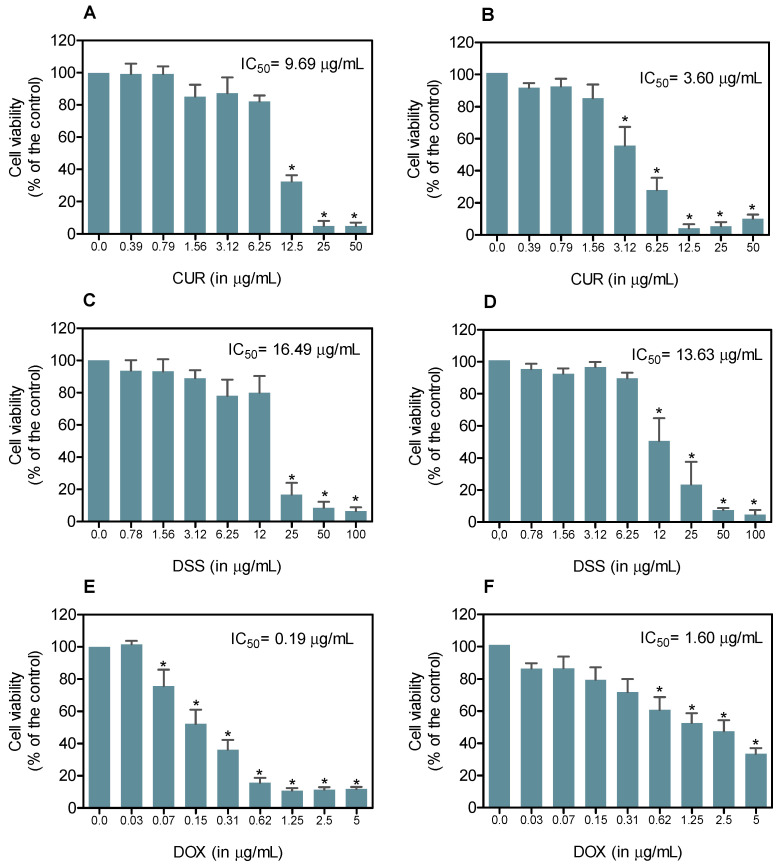
Effect of CUR and DSS on the viability of B16-F10 (**A**,**C**,**E**) and MRC-5 (**B**,**D**,**F**) cells measured by the Alamar Blue method after 72 h of incubation. DOX was used as a positive control. Data are shown as the mean ± S.E.M. of three independent experiments carried out in duplicate. * *p* < 0.05 compared with control (untreated cells) by ANOVA followed by the Student Newman-Keuls test.

CUR and DSS showed cytotoxic effects on B16-F10 and MRC-5 cells in a concentration-dependent manner after 72 h of incubation, as evaluated using the Alamar Blue method (Figure 1). The IC_50_ values found in B16-F10 cells were 9.69, 16.49, and 0.19 µg/mL for CUR, DSS, and DOX, respectively, while those in MRC-5 cells were 3.60, 13.63, and 1.60 µg/mL, respectively.

**Figure 2:**
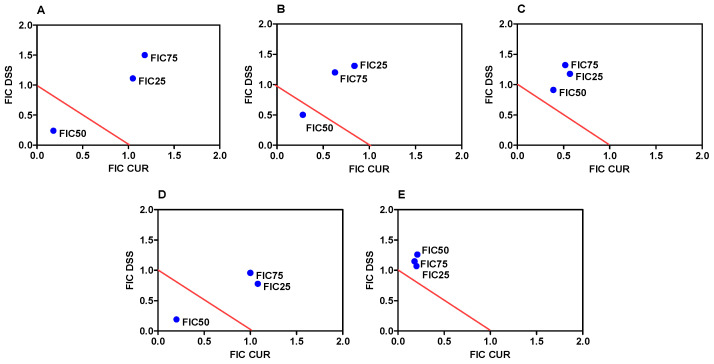
Isobolograms of the effects of the combination of CUR with DSS at ratios of 1:2 (**A**), 1:3 (**B**), 1:4 (**C**), 2:3 (**D**), and 1:10 (**E**) on the viability of B16-F10 cells. The fractional inhibitory concentration (FIC) values were calculated following the formula FIC(a) = effect (a) of the compound in combination/effect (a) of the compound alone, where (a) are the effects of 25%, 50%, and 75% inhibition resulting in FIC25, FIC50, and FIC75, respectively. Isobolograms were constructed using the coordinates formed by the FIC (CUR + DSS) of the 25%, 50%, and 75% effects. The line linking the number 1 in both axes was used. Points below this line indicate synergistic results of combination, and points above the line indicate antagonism. Points upon the line indicate an addictive effect.

Next, we tested the combination of CUR with DSS in five different ratios: 1:2, 1:3, 1:4, 2:3. and 1:10 (Figures S1–S5). Although these combinations were also toxic to noncancerous MRC-5 cells, enhanced cytotoxicity was observed in B16-F10 cells. The effect on B16-F10 cells was evaluated using FIC25, FIC50, and FIC75, revealing the magnitude of the concentration of each compound in relation to the same compound alone (Table S1). These data were also observed in isobolograms (Figure 2), where we found synergic effects for the combinations 1:2, 1:3, and 2:3 in FIC50. Therefore, these combinations were selected for further studies.

### 3.2. Combination Therapy with CUR and DSS Causes Apoptotic Cell Death in B16-F10 Melanoma Cells

In a new set of experiments, apoptosis quantification was evaluated in B16-F10 cells by annexin-V/PI double staining using flow cytometry after 48 and 72 h incubation. The numbers of viable (annexin-V/PI double-negative cells), apoptotic (all annexin-V-positive cells), and necrotic cells (annexin-V-negative/PI-positive cells) were quantified. The IC_50_ values of each compound were used (CUR 10 µg/mL and DSS 18 µg/mL). The ratios of 1:2 (CUR 2 µg/mL and DSS 4 µg/mL), 1:3 (CUR 2 µg/mL and DSS 6 µg/mL), and 2:3 (CUR 2 µg/mL and DSS 3 µg/mL) were also tested.

**Figure 3:**
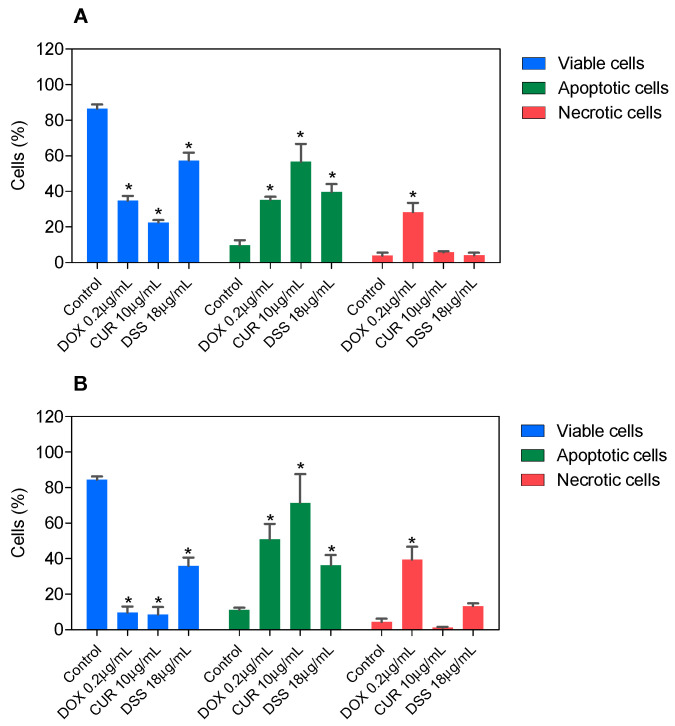
Effect of CUR and DSS on the viability of B16-F10 cells measured by annexin V-FITC/PI staining after 48 (**A**) and 72 (**B**) h of incubation. DOX was used as a positive control. Data are shown as the mean ± S.E.M. of three independent experiments carried out in duplicate. * *p* < 0.05 compared with control (0.5% DMSO) by ANOVA followed by Student Newman-Keuls test.

**Figure 4:**
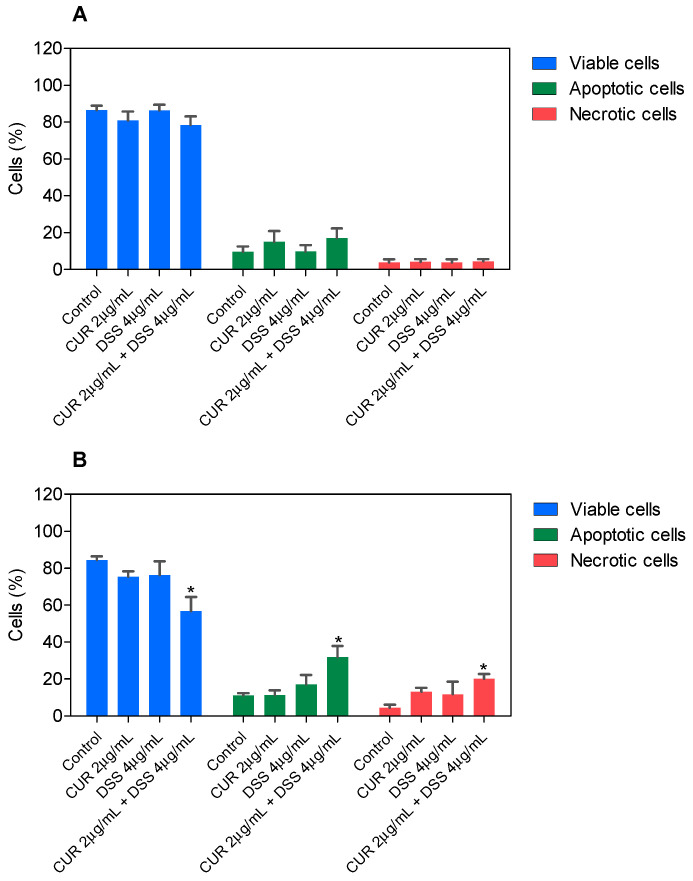
Effect of the combination of CUR with DSS at a ratio of 1:2 on the viability of B16-F10 cells measured by annexin V-FITC/PI staining after 48 (**A**) and 72 (**B**) h of incubation. Data are shown as the mean ± S.E.M. of three independent experiments carried out in duplicate. * *p* < 0.05 compared with control (0.5% DMSO) by ANOVA followed by Student Newman-Keuls test.

**Figure 5:**
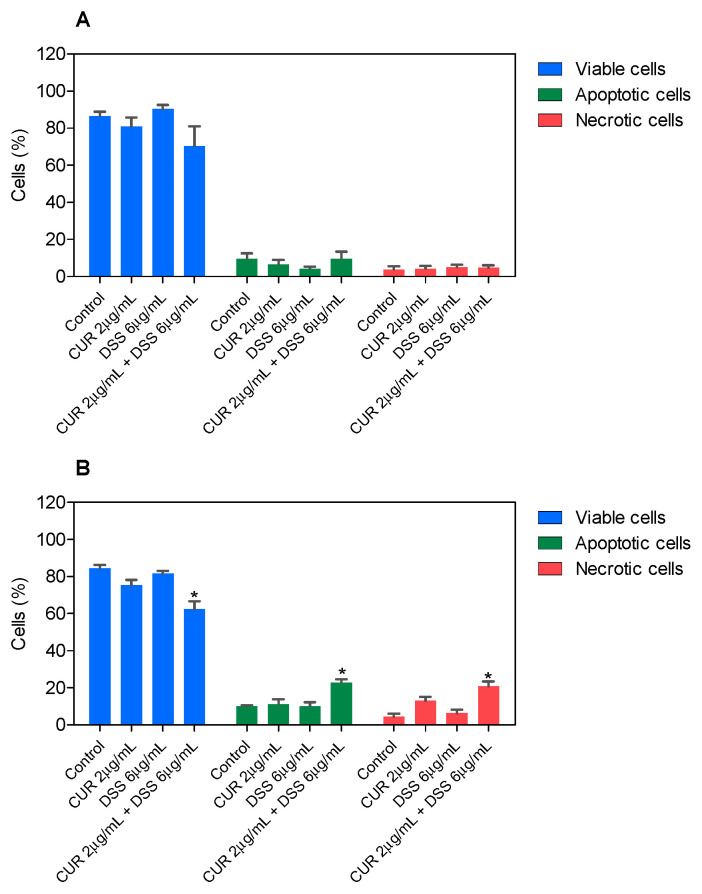
Effect of the combination of CUR with DSS at a ratio of 1:3 on the viability of B16-F10 cells measured by annexin V-FITC/PI staining after 48 (**A**) and 72 (**B**) h of incubation. Data are shown as the mean ± S.E.M. of three independent experiments carried out in duplicate. * *p* < 0.05 compared with control (0.5% DMSO) by ANOVA followed by Student Newman-Keuls test.

**Figure 6:**
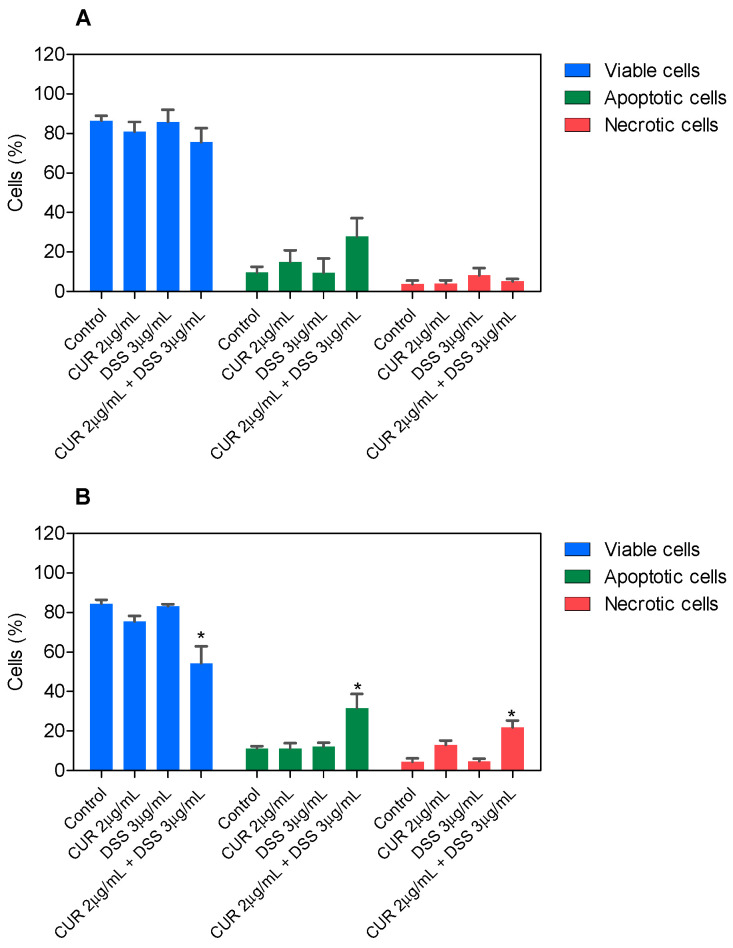
Effect of the combination of CUR with DSS at a ratio of 2:3 on the viability of B16-F10 cells measured by annexin V-FITC/PI staining after 48 (**A**) and 72 (**B**) h of incubation. Data are shown as the mean ± S.E.M. of three independent experiments carried out in duplicate. * *p* < 0.05 compared with control (0.5% DMSO) by ANOVA followed by Student Newman-Keuls test.

In B16-F10, CUR treatment led to 56.6% and 71.5% apoptosis after 48 and 72 h incubation, respectively, whereas DSS caused 39.7% and 36.3% apoptosis (Figure 3 and Figure S6). The combinations tested significantly increased apoptosis after 72 h of incubation. Combination 1:2 led to 32.0% apoptosis in B16-F10 cells (Figure 4 and Figure S7), while combination 1:3 caused 22.7% apoptosis (Figure 5 and Figure S8), and combination 2:3 caused 31.6% apoptosis (Figure 6 and Figure S9) after 72 h of incubation. A statistically significant proportional reduction in the viable cells was also observed.

**Figure 7:**
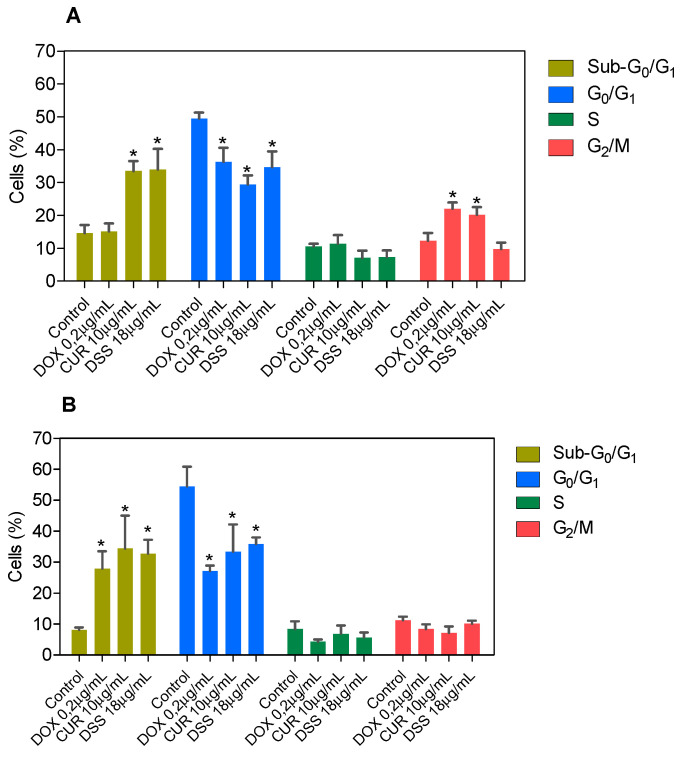
Effect of CUR and DSS on DNA fragmentation and cell cycle distribution of B16-F10 cells after 48 (**A**) and 72 (**B**) h of incubation. DOX was used as a positive control. Data are shown as the mean ± S.E.M. of three independent experiments carried out in duplicate. * *p* < 0.05 compared with control (0.5% DMSO) by ANOVA followed by Student Newman-Keuls test.

**Figure 8:**
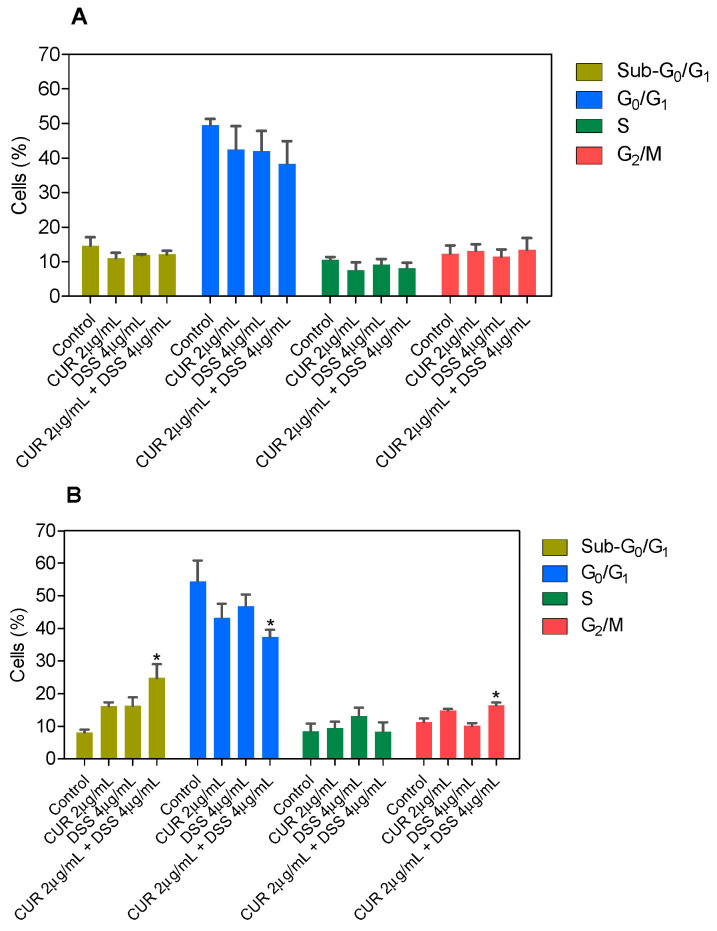
Effect of the combination of CUR with DSS at a ratio of 1:2 on DNA fragmentation and cell cycle distribution of B16-F10 cells after 48 (**A**) and 72 (**B**) h of incubation. Data are shown as the mean ± S.E.M. of three independent experiments carried out in duplicate. * *p* < 0.05 compared with control (0.5% DMSO) by ANOVA followed by Student Newman-Keuls test.

**Figure 9:**
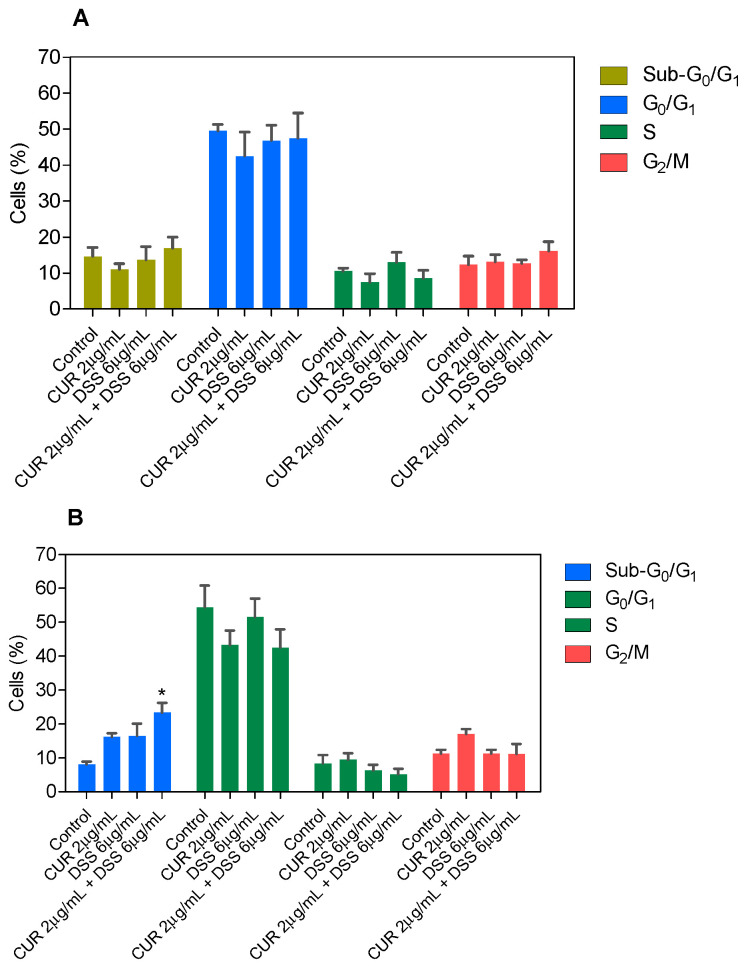
Effect of the combination of CUR with DSS at a ratio of 1:3 on DNA fragmentation and cell cycle distribution of B16-F10 cells after 48 (**A**) and 72 (**B**) h of incubation. Data are shown as the mean ± S.E.M. of three independent experiments carried out in duplicate. * *p* < 0.05 compared with control (0.5% DMSO) by ANOVA followed by Student Newman-Keuls test.

**Figure 10:**
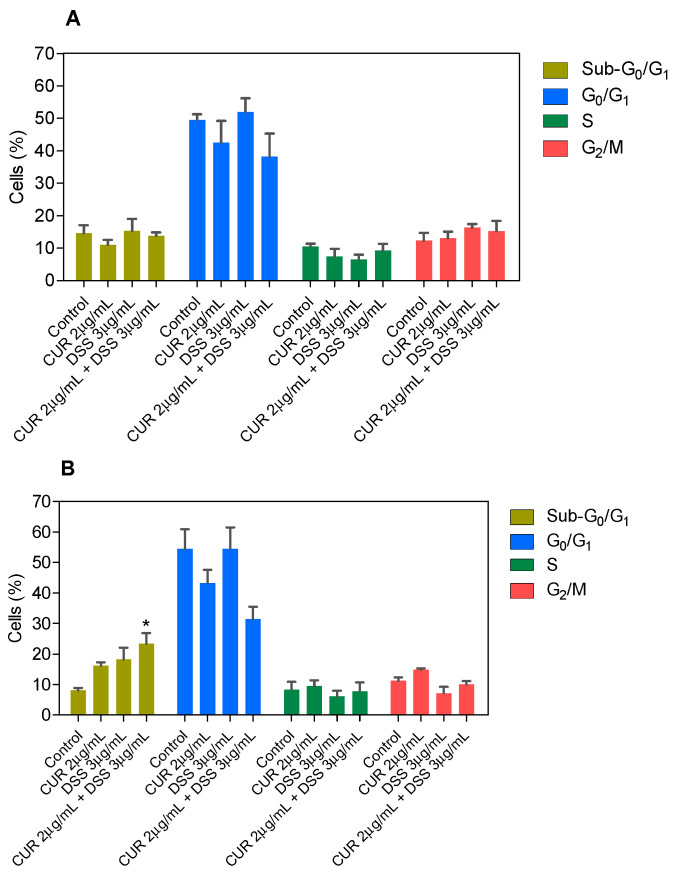
Effect of the combination of CUR with DSS at a ratio of 2:3 on DNA fragmentation and cell cycle distribution of B16-F10 cells after 48 (**A**) and 72 (**B**) h of incubation. Data are shown as the mean ± S.E.M. of three independent experiments carried out in duplicate. * *p* < 0.05 compared with control (0.5% DMSO) by ANOVA followed by Student Newman-Keuls test.

Next, the DNA content was measured by flow cytometry to quantify the internucleosomal DNA fragmentation and cell cycle distribution in B16-F10 cells treated with CUR and DSS alone or in combination after 48 and 72 h incubation. All DNA that was subdiploid (sub-G_0_/G_1_) was considered fragmented. CUR induced 33.5% and 34.4% DNA fragmentation in B16-F10 cells, while DSS caused 34.0% and 32.7% DNA fragmentation after 48 and 72 h of incubation, respectively (Figure 7 and Figure S10). After 72 h of incubation, the 1:2 combination induced 24.9% DNA fragmentation in B16-F10 cells (Figure 8 and Figure S11), while the 1:3 (Figure 9 and Figure S12) and 2:3 (Figure 10 and Figure S13) combinations caused 23.4% DNA fragmentation. No significant changes were observed after 48 h of incubation. A proportional reduction in the cell cycle phase was also observed. An increase in the cell cycle phase G_2_/M was found after 48 h of incubation with CUR, as well as in the combination 1:2 after 72 h of incubation. DOX caused cell cycle arrest at the G_2_/M phase, which was followed by DNA fragmentation in B16-F10 cells.

### 3.3. Combination Therapy with CUR and DSS Induces Oxidative Stress in B16-F10 Melanoma Cells

**Figure 11:**
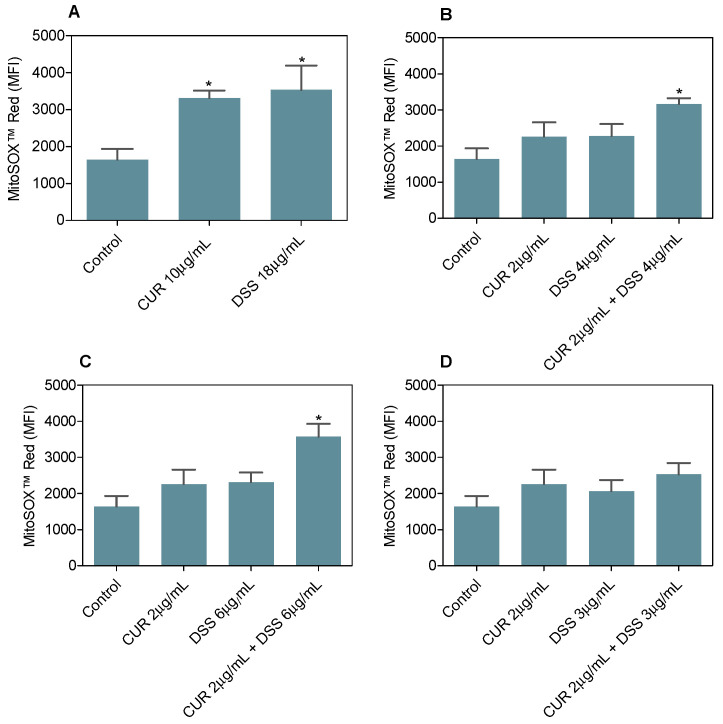
Effect of CUR and DSS (**A**) and their combinations at ratios of 1:2 (**B**), 1:3 (**C**), and 2:3 (**D**) on the mitochondrial superoxide level of B16-F10 cells measured by MitoSOX™ Red staining after 24 h of incubation. Data are shown as the mean ± S.E.M. of three independent experiments carried out in duplicate. * *p* < 0.05 compared with control (0.5% DMSO) by ANOVA followed by Student Newman-Keuls test. MFI = Mean Fluorescence Intensity.

MitoSOX™ Red was used to quantify mitochondrial superoxide levels in B16-F10 cells treated with CUR and DSS alone or in combination after 24 h of incubation (Figure 11). CUR or DSS significantly increased MitoSOX™ Red staining in B16-F10 cells, showing an MFI of 3313 ± 204.3 for CUR and 3532 ± 661.9 for DSS, against 1641 ± 292.6 found for the negative control, indicating increased oxidative stress. A significant increase in oxidative stress was also observed in B16-F10 cells treated with the combinations 1:2 (MFI of 3168 ± 156.7) and 1:3 (MFI of 3571 ± 360.1).

### 3.4. Combination Therapy with CUR and DSS Inhibits B16-F10 Melanoma Cells Grown In Vivo

**Figure 12:**
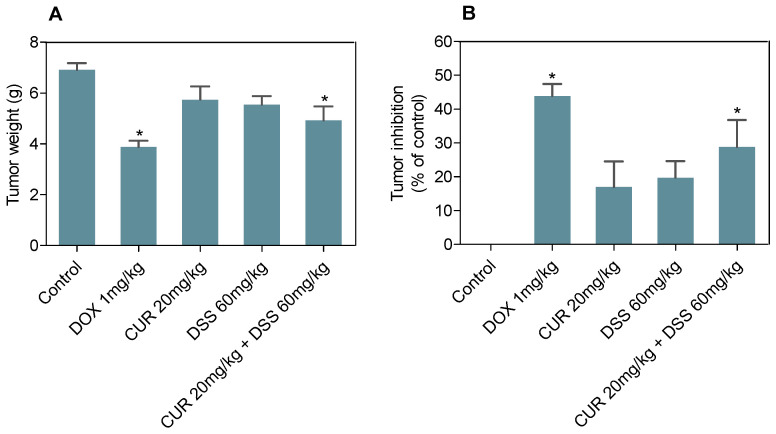
Effect of CUR and DSS and their combination at a ratio of 1:3 on the in vivo development of B16-F10 cells measured by tumor weight (**A**) and tumor inhibition (**B**). DOX was used as a positive control. Data are shown as the mean ± S.E.M. of 7–10 animals. * *p* < 0.05 compared with control (5% DMSO) by ANOVA followed by Student Newman-Keuls test.

The antitumor activities of CUR and DSS alone and in combination were evaluated in C57BL/6 mice bearing B16-F10 cells (Figure 12). The treatment was performed by intraperitoneal injection of 20 mg/kg CUR, 60 mg/kg DSS and their combination at a ratio of 1:3 (20 mg/kg CUR + 60 mg/kg DSS) for 15 days. DOX was used as a positive control at 1 mg/kg. At the end of treatment, the mean tumor mass weight of the control group was 6.9 ± 0.3 g. CUR and DSS showed mean tumor mass weights of 5.7 ± 0.5 and 5.6 ± 0.3 g, respectively, while an average tumor mass of 4.9 ± 0.6 g was found for the combination of CUR with DSS. Tumor mass inhibition rates were 17.0%, 19.8%, and 28.8% for CUR, DSS, and the combination, respectively. DOX (1 mg/kg) reduced tumor weight by 43.9%.

**Figure 13:**
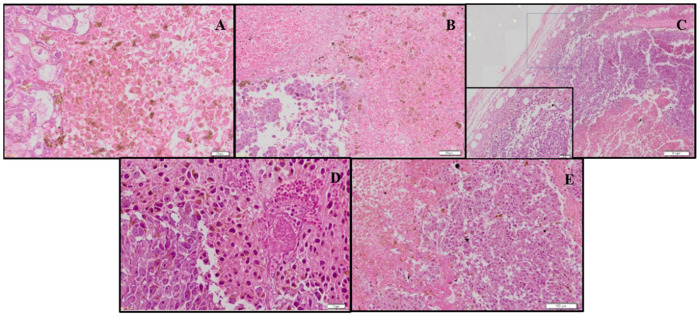
Representative histological analysis of B16-F10 tumor tissues stained with hematoxylin and eosin and analyzed by light microscopy. The animals were treated with 5% DMSO (**A**), 1 mg/kg DOX (**B**), 20 mg/kg CUR (**C**), 60 mg/kg DSS (**D**), or 20 mg/kg CUR + 60 mg/kg DSS (**E**). Bars = 20 µm (**A**,**D**), 50 µm (**B**), 200 µm (**C**), or 100 µm (**E**).

In the histological analysis of tumors (Figure 13), a highly proliferative tumor exhibiting rounded and disrupted cells was observed. Atypical mitosis, apoptosis, and necrosis were frequent features. In CUR-treated animals, we observed more delimitated and less vascularized tumors.

The systemic toxic effect of the CUR and DSS treatments or their combination was also evaluated. Three animals died in the combination group, and one animal died in each group, with the exception of the control group. No significant changes were found in the relative mass of the organs or body weights (Table S2). In the hematological parameter analyses, an increase in erythrocytes, hemoglobin, MCV, and platelets was found in the groups treated with DOX and DSS compared to the control (Table S3).

The histopathological examinations of hearts and kidneys showed well-preserved structures in all experimental groups (Figures S14 and S15). In the liver, areas of hydropic degeneration, vascular hyperemia and inflammation were frequent in the control groups, although swelling of hepatocytes was seen in the CUR and DSS groups (Figure S16). Single treatment with CUR or DSS resulted in mild inflammation and areas of fibrosis in the kidney and liver. In the combination treatment group, mild edema was identified. Focal areas of microgoticular steatosis were observed in some animals of the control and groups treated with DOX, CUR, and the combination treatment. In the lungs, areas of inflammation, vascular hyperemia, and alveolar septal thickening were observed in all groups (Figure S17).

## 4. Discussion

The treatment for patients with melanoma usually shows low response rates associated with the development of resistance and side effects with low overall survival [1,2,3,4,5,6]. In this work, we demonstrated for the first time that CUR and DSS inhibit the in vitro and in vivo development of melanoma B16-F10 cells and, when combined, presented synergistic action. Induction of apoptosis and oxidative stress was also found.

Repositioning clinically approved drugs has been considered a viable approach to building new anti-cancer drugs. The repositioning allows prior knowledge of safety factors, bioavailability, and formulations, offering advantages such as shorter development time and lower research costs, providing agility in accessing new therapeutic options for cancer patients [39,40]. The time between new drug development and clinical trials averages 9 years, with a success rate of less than 10% and an average patient cost per drug of several hundred million dollars. In contrast, drug repositioning can take 3 to 4 years for clinical trials and costs only a fraction of the amounts needed to test a new class of drug in patients [41].

CUR is a nutraceutical drug, and DSS is an FDA-approved drug, both of which are used long-term by humans. This indicates that their combination can be safe, even if it has an effect on noncancerous MRC-5 cells. Nevertheless, drug repositioning may be accompanied by side effects that have not been previously identified and described [42]. Therefore, more experiments are required to validate the safety of the CUR and DSS combination.

Apoptosis has been reported as a mechanism of programmed cell death in the presence of cytotoxic agents with chemotherapeutic potential. Treatment with high concentrations of CUR has been described as capable of inducing apoptosis, depending on the cell and tissue type, by both extrinsic and intrinsic pathways, as well as by increased endoplasmic reticulum stress [43]. The intrinsic induction of apoptosis by CUR is activated in response to cellular signals, including stress or DNA damage [34,44]. DSS is responsible for activating the extrinsic pathway of apoptosis [35]. In this work, we showed that B16-F10 cells treated with the combination of CUR with DSS for a period of 72 h showed increased externalization of phosphatidylserine, suggesting cell death by apoptosis.

Considering that the redox mechanism of melanocytes is extremely important for tumor progression, our result here was obtained by the combination of the pro-oxidant profile, favored by the use of CUR, and by the antagonistic action of the antioxidant system performed by DSS, resulting in increased oxidative stress in tumor cells, which was confirmed by the presence of superoxide radicals in B16-F10 cells. In addition, other targets, including the inhibition of the ubiquitin-proteasome system, were reported for these compounds and may contribute to their cytotoxicity [45,46].

The antitumor activities of CUR have been demonstrated in melanoma cells [15] and animal models [47]. DSS has been shown to exert protective effects on organs in experimental studies, preventing myocardial damage [48], in addition to sensitizing tumor cells to radiotherapy and increasing the cytotoxicity of antineoplastic drugs, which can be used as adjuvant therapy [49]. In our in vivo experiment, we also observed that lower doses of the combined drugs led to a significant reduction in the progression of B16F10 cells in mice.

This study provided data on the potential cytotoxic activity of the combination of CUR with DSS and may provide a useful tool for the development of novel therapeutic combinations against melanoma.

## Data Availability

Data will be made available upon reasonable request.

## Supplementary Materials

The following supporting information can be downloaded at: https://www.mdpi.com/article/10.3390/biom12111600/s1, Figure S1. Effect of the combination of CUR and DSS at a ratio of 1:2 on the viability of B16-F10 (A) and MRC-5 (B) cells measured by the alamar blue method after 72 h of incubation. Figure S2. Effect of the combination of CUR and DSS at a ratio of 1:3 on the viability of B16-F10 (A) and MRC-5 (B) cells measured by the alamar blue method after 72 h of incubation. Figure S3. Effect of the combination of CUR and DSS at a ratio of 1:4 on the viability of B16-F10 (A) and MRC-5 (B) cells measured by the alamar blue method after 72 h of incubation. Figure S4. Effect of the combination of CUR and DSS at a ratio of 2:3 on the viability of B16-F10 (A) and MRC-5 (B) cells measured by the alamar blue method after 72 h of incubation. Figure S5. Effect of the combination of CUR and DSS at a ratio of 1:10 on the viability of B16-F10 (A) and MRC-5 (B) cells measured by the alamar blue method after 72 h of incubation. Figure S6. Representative dotplots of the effect of CUR and DSS on the viability of B16-F10 cells measured by annexin V-FITC/PI staining. Figure S7. Representative dotplots of the effect of the combination of CUR with DSS at a ratio of 1:2 on the viability of B16-F10 cells measured by annexin V-FITC/PI staining. Figure S8. Representative dotplots of the effect of the combination of CUR with DSS at a ratio of 1:3 on the viability of B16-F10 cells measured by annexin V-FITC/PI staining. Figure S9. Representative dotplots of the effect of the combination of CUR with DSS at a ratio of 2:3 on the viability of B16-F10 cells measured by annexin V-FITC/PI staining. Figure S10. Representative histograms of the effect of CUR and DSS on DNA fragmentation and cell cycle distribution of B16-F10 cells. Figure S11. Representative histograms of the effect of the combination of CUR with DSS at a ratio of 1:2 on DNA fragmentation and cell cycle distribution of B16-F10 cells. Figure S12. Representative histograms of the effect of the combination of CUR with DSS at a ratio of 1:3 on DNA fragmentation and cell cycle distribution of B16-F10 cells. Figure S13. Representative histograms of the effect of the combination of CUR with DSS at a ratio of 2:3 on DNA fragmentation and cell cycle distribution of B16-F10 cells. Figure S14. Representative histological analysis of hearts stained with hematoxylin and eosin and analyzed by light microscopy. Figure S15. Representative histological analysis of kidneys stained with hematoxylin and eosin and analyzed by light microscopy. Figure S16. Representative histological analysis of livers stained with hematoxylin and eosin and analyzed by light microscopy. Figure S17. Representative histological analysis of lungs stained with hematoxylin and eosin and analyzed by light microscopy. Table S1. Inhibitory concentrations of CUR and DSS alone and in combination. Table S2. Effect of CUR and DSS and their combination on body and relative organ weight from C57BL/6 mice bearing B16-F10 cells. Table S3. Effect of CUR and DSS and their combination on hematological parameters of peripheral blood from C57BL/6 mice bearing B16-F10 cells.

## References

[1] Miller A.J., Mihm M.C. (2006). Melanoma. N. Engl. J. Med..

[2] Wagstaff W., Mwamba R.N., Grullon K., Armstrong M., Zhao P., Hendren-Santiago B., Qin K.H., Li A.J., Hu D.A., Youssef A. (2022). Melanoma: Molecular genetics, metastasis, targeted therapies, immunotherapies, and therapeutic resistance. Genes Dis..

[3] Davis L.E., Shalin S.C., Tackett A.J. (2019). Current state of melanoma diagnosis and treatment. Cancer Biol. Ther..

[4] Sung H., Ferlay J., Siegel R.L., Laversanne M., Soerjomataram I., Jemal A., Bray F. (2021). Global Cancer Statistics 2020: GLOBOCAN Estimates of Incidence and Mortality Worldwide for 36 Cancers in 185 Countries. CA Cancer J. Clin..

[5] Maio M., Grob J.J., Aamdal S., Bondarenko I., Robert C., Thomas L., Garbe C., Chiarion-Sileni V., Testori A., Chen T.T. (2015). Five-year survival rates for treatment-naive patients with advanced melanoma who received ipilimumab plus dacarbazine in a phase III trial. J. Clin. Oncol..

[6] Hao M., Song F., Du X., Wang G., Yang Y., Chen K., Yang J. (2015). Advances in targeted therapy for unresectable melanoma: New drugs and combinations. Cancer Lett..

[7] Obrador E., Liu-Smith F., Dellinger R.W., Salvador R., Meyskens F.L., Estrela J.M. (2019). Oxidative stress and antioxidants in the pathophysiology of malignant melanoma. Biol. Chem..

[8] Kim E., Panzella L., Napolitano A., Payne G.F. (2020). Redox Activities of melanins investigated by electrochemical reverse engineering: Implications for their roles in oxidative stress. J. Investig. Dermatol..

[9] Liu-Smith F., Dellinger R., Meyskens F.L. (2014). Updates of reactive oxygen species in melanoma etiology and progression. Arch. Biochem. Biophys..

[10] Ghasemi F., Shafiee M., Banikazemi Z., Pourhanifeh M.H., Khanbabaei H., Shamshirian A., Amiri Moghadam S., ArefNezhad R., Sahebkar A., Avan A. (2019). Curcumin inhibits NF-kB and Wnt/β-catenin pathways in cervical cancer cells. Pathol. Res. Pract..

[11] Sarkar F.H., Li Y., Wang Z., Padhye S. (2010). Lesson learned from nature for the development of novel anti-cancer agents: Implication of isoflavone, curcumin, and their synthetic analogs. Curr. Pharm. Des..

[12] Thayyullathil F., Chathoth S., Hago A., Patel M., Galadari S. (2008). Rapid reactive oxygen species (ROS) generation induced by curcumin leads to caspase-dependent and -independent apoptosis in L929 cells. Free Radic. Biol. Med..

[13] Sánchez Y., Simón G.P., Calviño E., de Blas E., Aller P. (2010). Curcumin stimulates reactive oxygen species production and potentiates apoptosis induction by the antitumor drugs arsenic trioxide and lonidamine in human myeloid leukemia cell lines. J. Pharmacol. Exp. Ther..

[14] Khan M.A., Gahlot S., Majumdar S. (2012). Oxidative stress induced by curcumin promotes the death of cutaneous T-cell lymphoma (HuT-78) by disrupting the function of several molecular targets. Mol. Cancer Ther..

[15] Liao W., Xiang W., Wang F.F., Wang R., Ding Y. (2017). Curcumin inhibited growth of human melanoma A375 cells via inciting oxidative stress. Biomed. Pharmacother..

[16] Watson J.L., Hill R., Yaffe P.B., Greenshields A., Walsh M., Lee P.W., Giacomantonio C.A., Hoskin D.W. (2010). Curcumin causes superoxide anion production and p53-independent apoptosis in human colon cancer cells. Cancer Lett..

[17] Gessner P.K., Gessner T. (2012). Disulfiram and Its Metabolite, Diethyldithiocarbamate: Pharmacology and Status in the Treatment of Alcoholism, Hiv Infections, Aids and Heavy Metal Toxicity.

[18] Wang N.N., Wang L.H., Li Y., Fu S.Y., Xue X., Jia L.N., Yuan X.Z., Wang Y.T., Tang X., Yang J.Y. (2018). Targeting ALDH2 with disulfiram/copper reverses the resistance of cancer cells to microtubule inhibitors. Exp. Cell Res..

[19] Guo F., Yang Z., Kulbe H., Albers A.E., Sehouli J., Kaufmann A.M. (2019). Inhibitory effect on ovarian cancer ALDH+ stem-like cells by Disulfiram and Copper treatment through ALDH and ROS modulation. Biomed. Pharmacother..

[20] Fruehauf J.P., Trapp V. (2008). Reactive oxygen species: An Achilles’ heel of melanoma?. Expert Rev. Anticancer Ther..

[21] Lopes-Rodrigues V., Sousa E., Vasconcelos M.H. (2016). Curcumin as a modulator of P-glycoprotein in cancer: Challenges and perspectives. Pharmaceuticals.

[22] Ekinci E., Rohondia S., Khan R., Dou Q.P. (2019). Repurposing disulfiram as an anti-cancer agent: Updated review on literature and patents. Recent Pat. Anticancer Drug Discov..

[23] Jangra A., Choi S.A., Yang J., Koh E.J., Phi J.H., Lee J.Y., Wang K.C., Kim S.K. (2020). Disulfiram potentiates the anticancer effect of cisplatin in atypical teratoid/rhabdoid tumors (AT/RT). Cancer Lett..

[24] Wang L., Yu Y., Zhou C., Wan R., Li Y. (2022). Anticancer effects of disulfiram: A systematic review of in vitro, animal, and human studies. Syst. Rev..

[25] Sukprasansap M., Chanvorachote P. (2022). Evidence of potential plant-derived compounds with anticancer effects on lung cancer: Clinical and molecular pharmacology approaches. Anticancer Res..

[26] Ng C.X., Affendi M.M., Chong P.P., Lee S.H. (2022). The potential of plant-derived extracts and compounds to augment anticancer effects of chemotherapeutic drugs. Nutr. Cancer.

[27] Hussain Y., Islam L., Khan H., Filosa R., Aschner M., Javed S. (2021). Curcumin-cisplatin chemotherapy: A novel strategy in promoting chemotherapy efficacy and reducing side effects. Phytother Res..

[28] Almeida-Silva J., Menezes D.S., Fernandes J.M.P., Almeida M.C., Vasco-Dos-Santos D.R., Saraiva R.M., Viçosa A.L., Perez S.A.C., Andrade S.G., Suarez-Fontes A.M. (2022). The repositioned drugs disulfiram/diethyldithiocarbamate combined to benznidazole: Searching for Chagas disease selective therapy, preventing toxicity and drug resistance. Front. Cell Infect. Microbiol..

[29] López-Lázaro M. (2008). Anticancer and carcinogenic properties of curcumin: Considerations for its clinical development as a cancer chemopreventive and chemotherapeutic agent. Mol. Nutr. Food Res..

[30] Ahmed S.A., Gogal R.M., Walsh J.E. (1994). A new rapid and simple non-radioactive assay to monitor and determine the proliferation of lymphocytes: An alternative to [3H]-thymidine incorporation assay. J. Immunol. Methods.

[31] Santos L.S., Silva V.R., Menezes L.R.A., Soares M.B.P., Costa E.V., Bezerra D.P. (2017). Xylopine induces oxidative stress and causes G2/M phase arrest, triggering caspase-mediated apoptosis by p53-independent pathway in HCT116 cells. Oxid. Med. Cell Longev..

[32] Silva V.R., Correa R.S., Santos L.S., Soares M.B.P., Batista A.A., Bezerra D.P. (2018). A ruthenium-based 5-fluorouracil complex with enhanced cytotoxicity and apoptosis induction action in HCT116 cells. Sci. Rep..

[33] Bill M.A., Bakan C., Benson D.M., Fuchs J., Young G., Lesinski G.B. (2009). Curcumin induces proapoptotic effects against human melanoma cells and modulates the cellular response to immunotherapeutic cytokines. Mol. Cancer Ther..

[34] Kocyigit A., Guler E.M. (2017). Curcumin induce DNA damage and apoptosis through generation of reactive oxygen species and reducing mitochondrial membrane potential in melanoma cancer cells. Cell Mol. Biol..

[35] Morrison B.W., Doudican N.A., Patel K.R., Orlow S.J. (2010). Disulfiram induces copper-dependent stimulation of reactive oxygen species and activation of the extrinsic apoptotic pathway in melanoma. Melanoma Res..

[36] Nicoletti I., Migliorati G., Pagliaccim M.C., Grignanim F., Riccardi C. (1991). A rapid and simple method for measuring thymocyte apoptosis by propidium iodide staining and flow cytometry. J. Immunol. Methods.

[37] Rodrigues A.C., Bomfim L.M., Neves S.P., Menezes L.R., Dias R.B., Soares M.B., Prata A.P., Rocha C.A., Costa E.V., Bezerra D.P. (2015). Antitumor properties of the essential oil from the leaves of *Duguetia gardneriana*. Planta Med..

[38] Chou T.C. (2006). Theoretical basis, experimental design, and computerized simulation of synergism and antagonism in drug combination studies. Pharmacol. Rev..

[39] Grammer A.C., Lipsky P.E. (2017). Drug repositioning strategies for the identification of novel therapies for rheumatic autoimmune inflammatory diseases. Rheum. Dis. Clin. N. Am..

[40] Yang B., Shi J. (2020). Developing new cancer nanomedicines by repurposing old drugs. Angew. Chem. Int. Ed. Engl..

[41] Nowak-Sliwinska P., Scapozza L., Ruiz I.A.A. (2019). Drug repurposing in oncology: Compounds, pathways, phenotypes and computational approaches for colorectal cancer. Biochim. Biophys. Acta Rev. Cancer.

[42] Antoszczak M., Markowska A., Markowska J., Huczyński A. (2020). Old wine in new bottles: Drug repurposing in oncology. Eur. J. Pharmacol..

[43] Laubach V., Kaufmann R., Bernd A. (2019). Extrinsic or intrinsic apoptosis by curcumin and light: Still a mystery. Int. J. Mol. Sci..

[44] Mortezaee K., Salehi E., Mirtavoos-Mahyari H., Motevaseli E., Najafi M., Farhood B., Rosengren R.J., Sahebkar A. (2019). Mechanisms of apoptosis modulation by curcumin: Implications for cancer therapy. J. Cell Physiol..

[45] Meraz-Torres F., Plöger S., Garbe C., Niessner H., Sinnberg T. (2020). Disulfiram as a therapeutic agent for metastatic malignant melanoma-old myth or new logos?. Cancers.

[46] Banerjee S., Ji C., Mayfield J.E., Goel A., Xiao J., Dixon J.E., Guo X. (2018). Ancient drug curcumin impedes 26S proteasome activity by direct inhibition of dual-specificity tyrosine-regulated kinase 2. Proc. Natl. Acad. Sci. USA.

[47] Odot J., Albert P., Carlier A., Tarpin M., Devy J., Madoulet C. (2004). In vitro and in vivo anti-tumoral effect of curcumin against melanoma cells. Int. J. Cancer.

[48] Sonawane V.K., Mahajan U.B., Shinde S.D., Chatterjee S., Chaudhari S.S., Bhangale H.A., Ojha S., Goyal S.N., Kundu C.N., Patil C.R. (2018). A chemosensitizer drug: Disulfiram prevents doxorubicin-induced cardiac dysfunction and oxidative stress in rats. Cardiovasc. Toxicol..

[49] Jiao Y., Hannafon B.N., Ding W.Q. (2016). Disulfiram’s anticancer activity: Evidence and mechanisms. Anticancer Agents Med. Chem..

