# Supplementary material for: Combination Therapy of Curcumin and Disulfiram Synergistically Inhibits the Growth of B16-F10 Melanoma Cells by Inducing Oxidative Stress
Source: Biomolecules. 2022 Oct 31;12(11):1600. doi: 10.3390/biom12111600 (PMC9687191; doi:10.3390/biom12111600)
Supplement: Supplementary file 1 [file biomolecules-12-01600-s001.zip › biomolecules-1965067-supplementary.pdf]

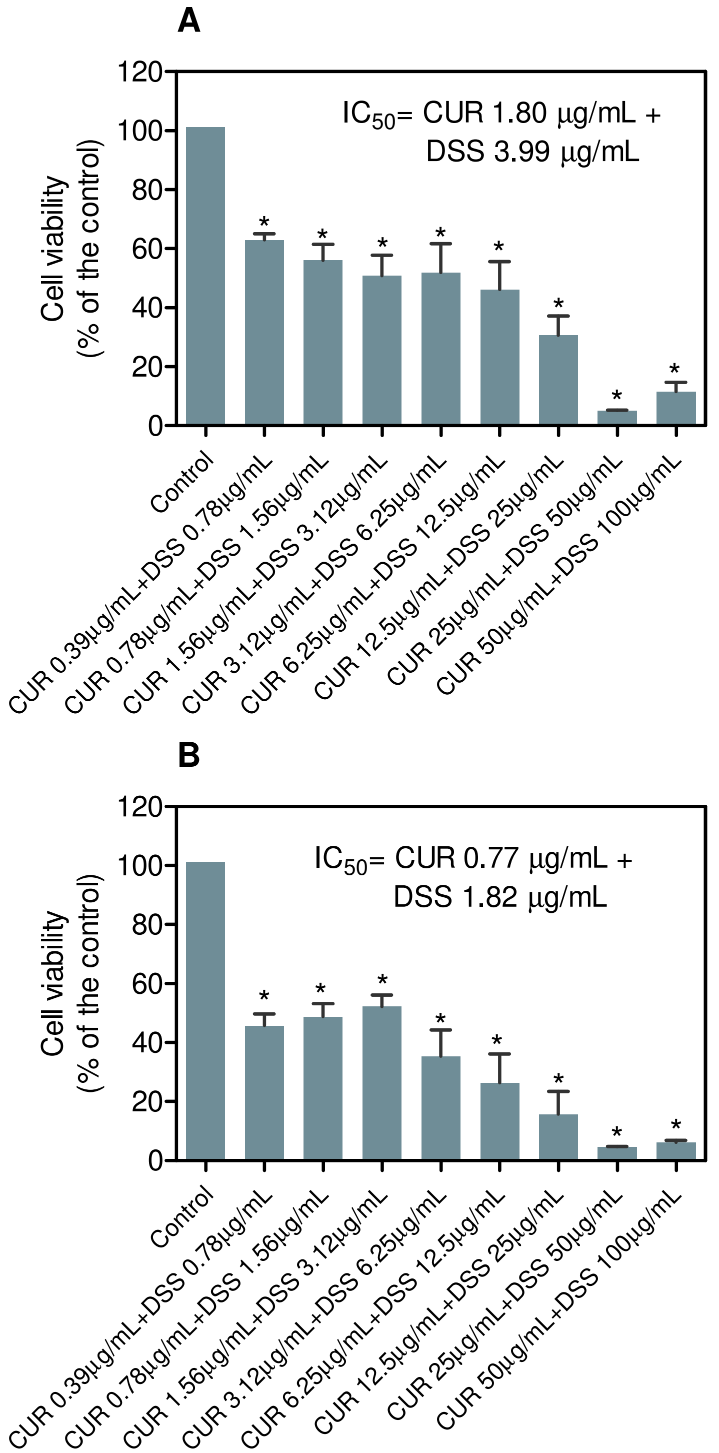


**Figure S1.** Effect of the combination of CUR and DSS at a ratio of 1:2 on the viability of B16-F10 (**A**) and MRC-5 (**B**) cells measured by the alamar blue method after 72 h of incubation. Data are shown as the mean ± S.E.M. of at least three independent experiments carried out in duplicate. *$P < 0.05$ compared with control (untreated cells) by ANOVA followed by Student Newman–Keuls test.

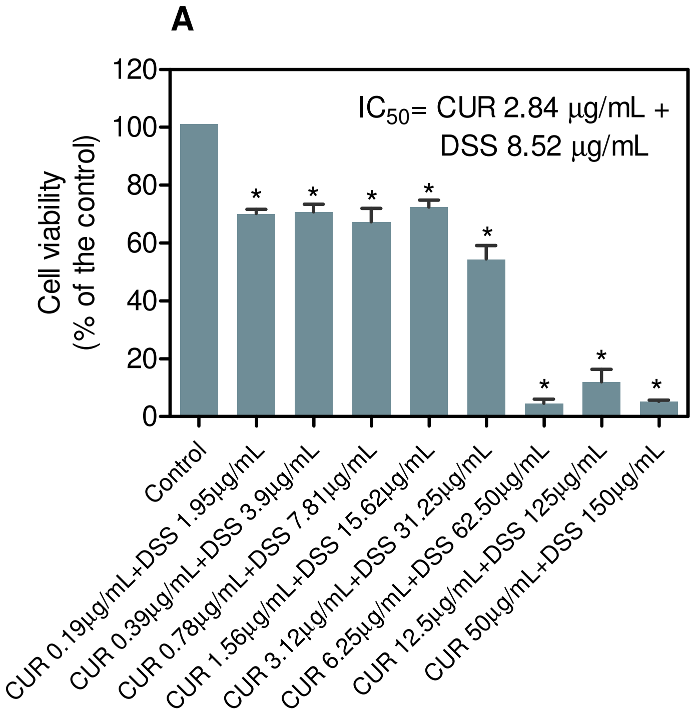


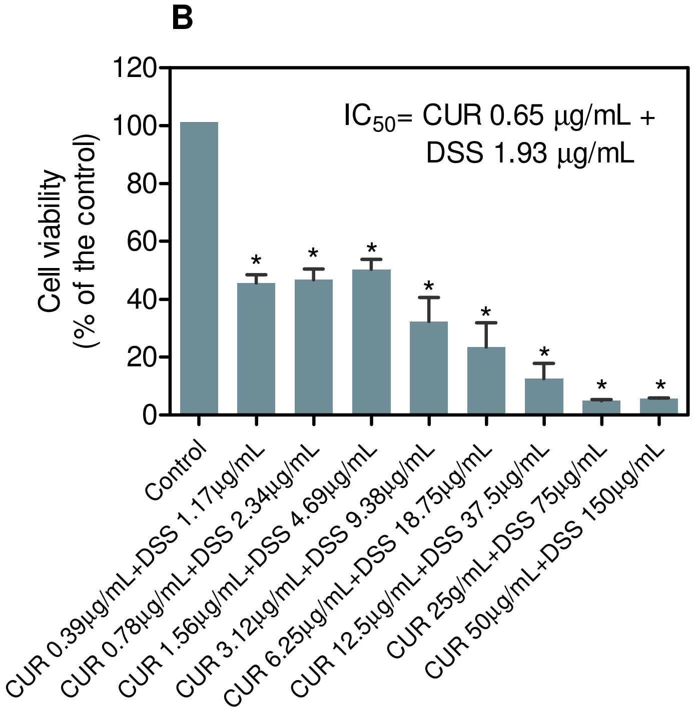


**Figure S2.** Effect of the combination of CUR and DSS at a ratio of 1:3 on the viability of B16-F10 (**A**) and MRC-5 (**B**) cells measured by the alamar blue method after 72 h of incubation. Data are shown as the mean ± S.E.M. of at least three independent experiments carried out in duplicate. *$P < 0.05$ compared with control (untreated cells) by ANOVA followed by Student Newman–Keuls test.

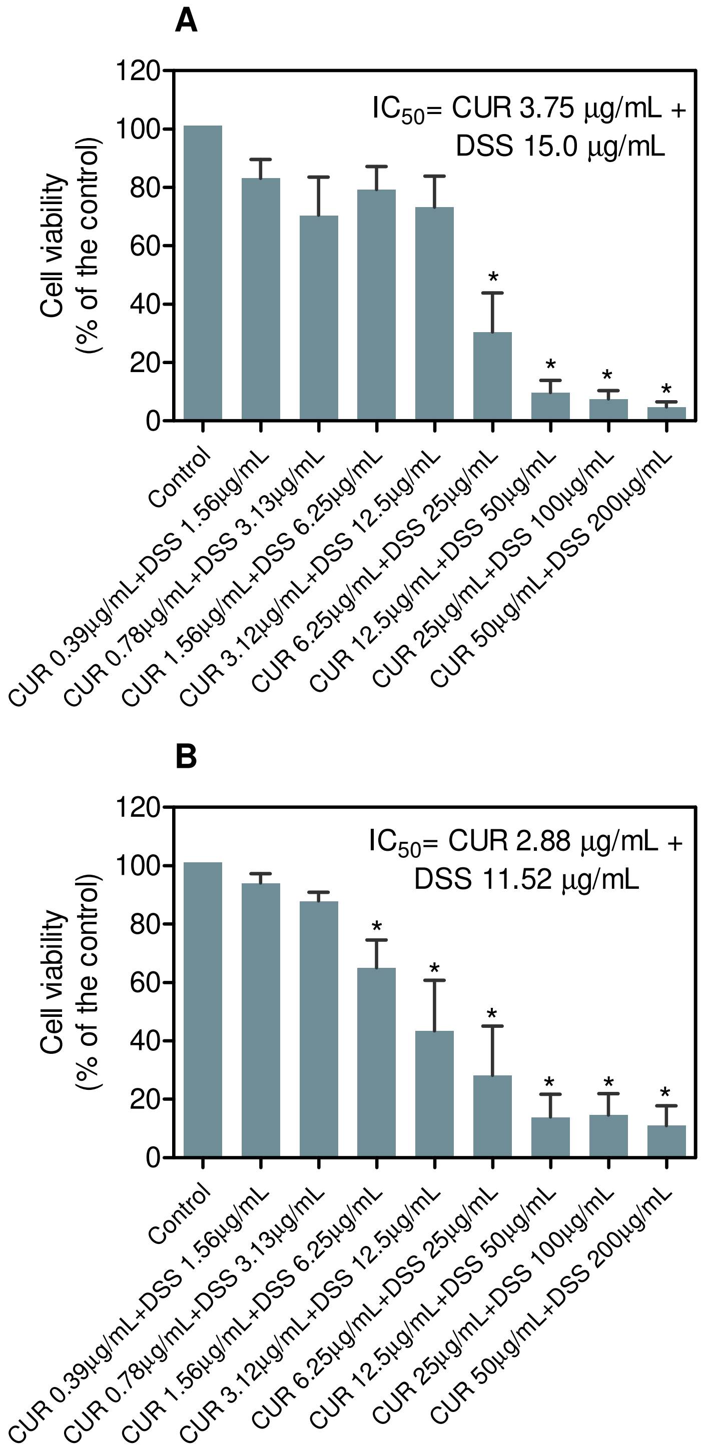


**Figure S3.** Effect of the combination of CUR and DSS at a ratio of 1:4 on the viability of B16-F10 (**A**) and MRC-5 (**B**) cells measured by the alamar blue method after 72 h of incubation. Data are shown as the mean ± S.E.M. of at least three independent experiments carried out in duplicate. *$P < 0.05$ compared with control (untreated cells) by ANOVA followed by Student Newman–Keuls test.

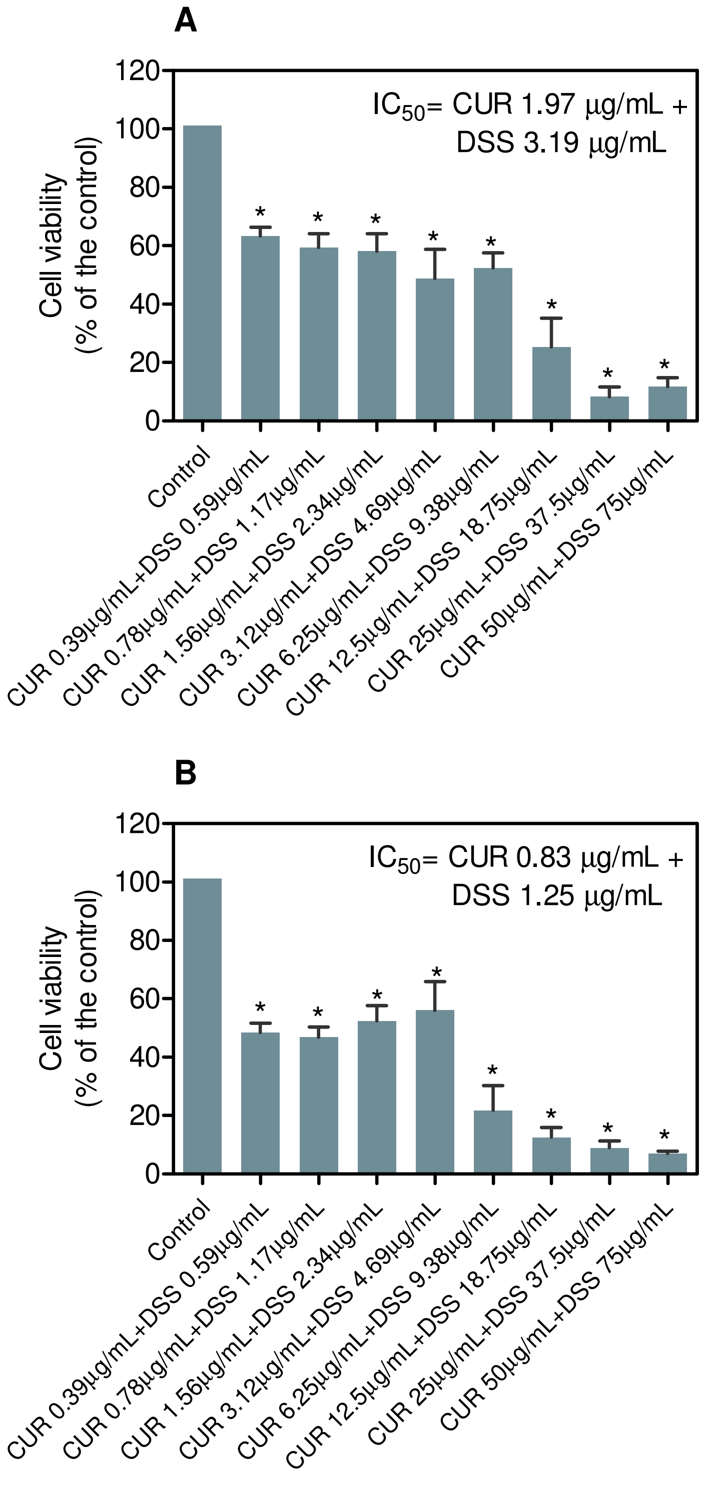


**Figure S4.** Effect of the combination of CUR and DSS at a ratio of 2:3 on the viability of B16-F10 (**A**) and MRC-5 (**B**) cells measured by the alamar blue method after 72 h of incubation. Data are shown as the mean ± S.E.M. of at least three independent experiments carried out in duplicate. *$P < 0.05$ compared with control (untreated cells) by ANOVA followed by Student Newman–Keuls test.

**A**

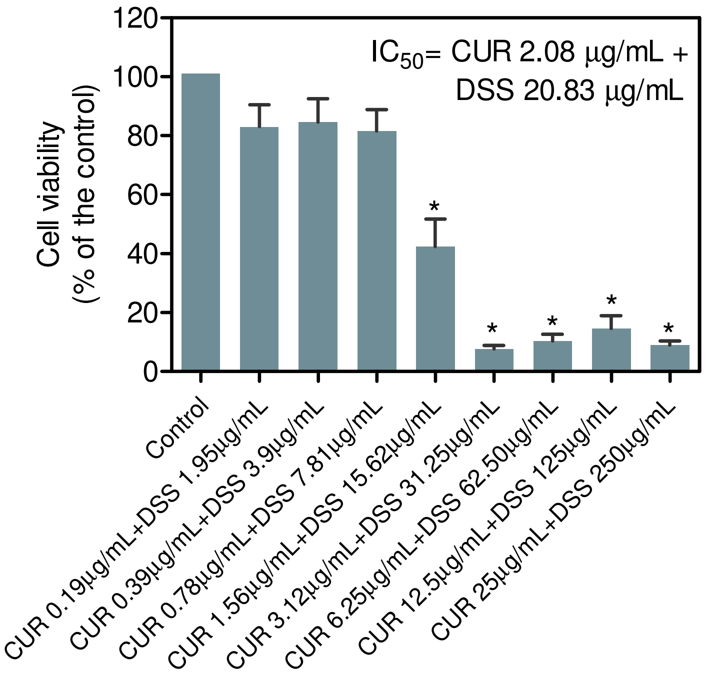


**B**

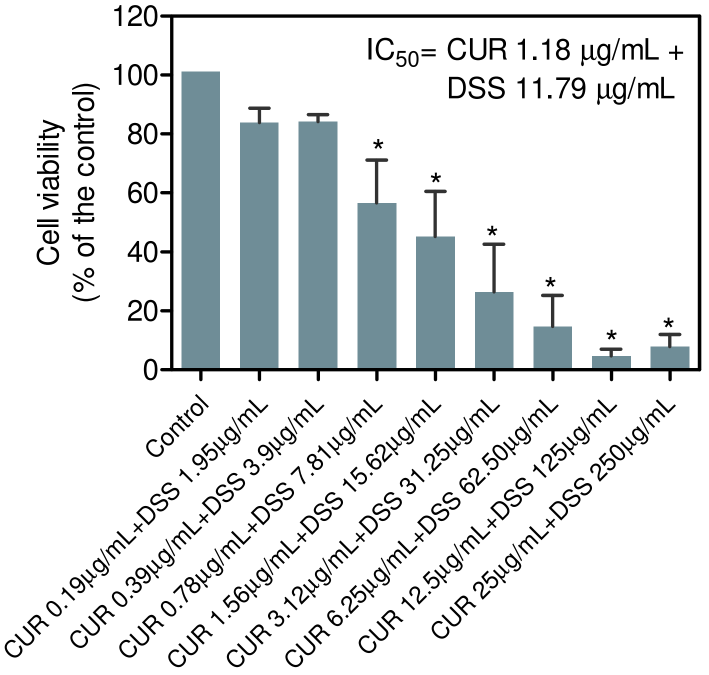


**Figure S5.** Effect of the combination of CUR and DSS at a ratio of 1:10 on the viability of B16-F10 (**A**) and MRC-5 (**B**) cells measured by the alamar blue method after 72 h of incubation. Data are shown as the mean ± S.E.M. of at least three independent experiments carried out in duplicate. *$P < 0.05$ compared with control (untreated cells) by ANOVA followed by Student Newman–Keuls test.

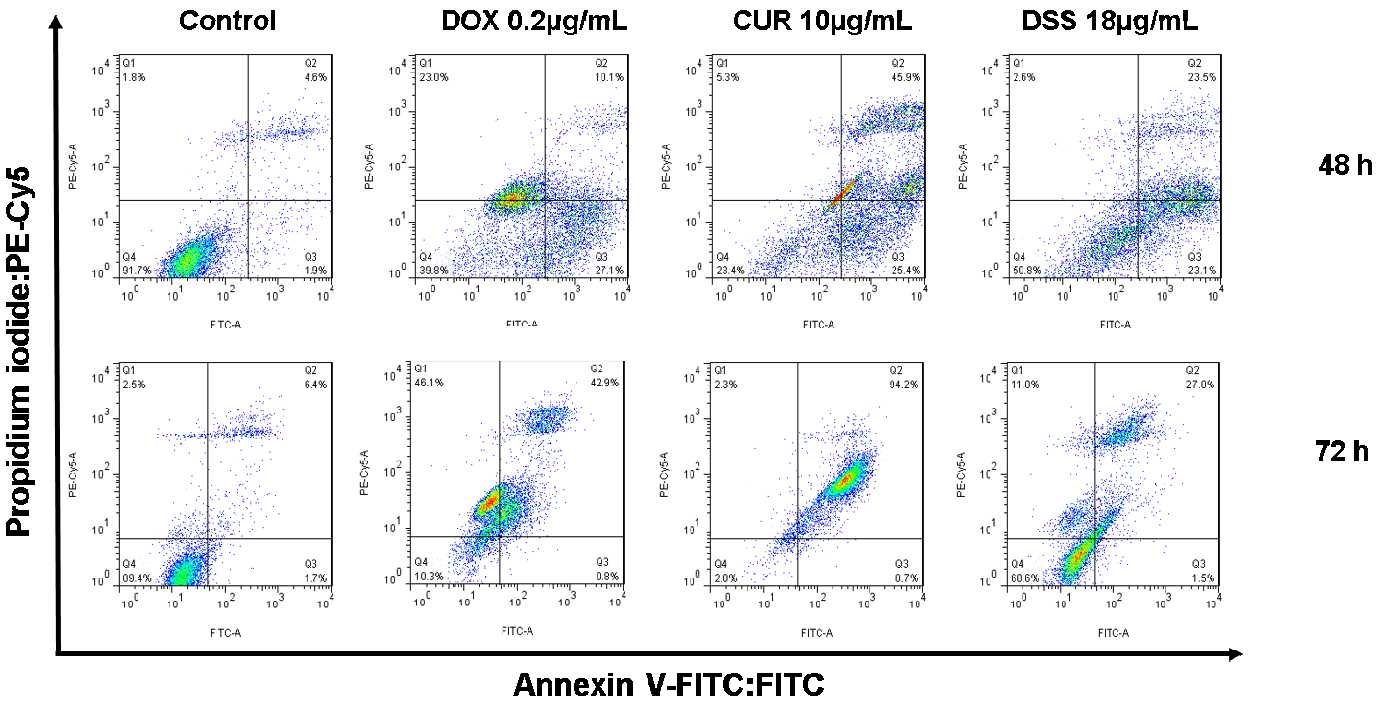


**Figure S6.** Representative dotplots of the effect of CUR and DSS on the viability of B16-F10 cells measured by annexin V-FITC/PI staining. DOX was used as a positive control, and the negative control received 0.5% DMSO.

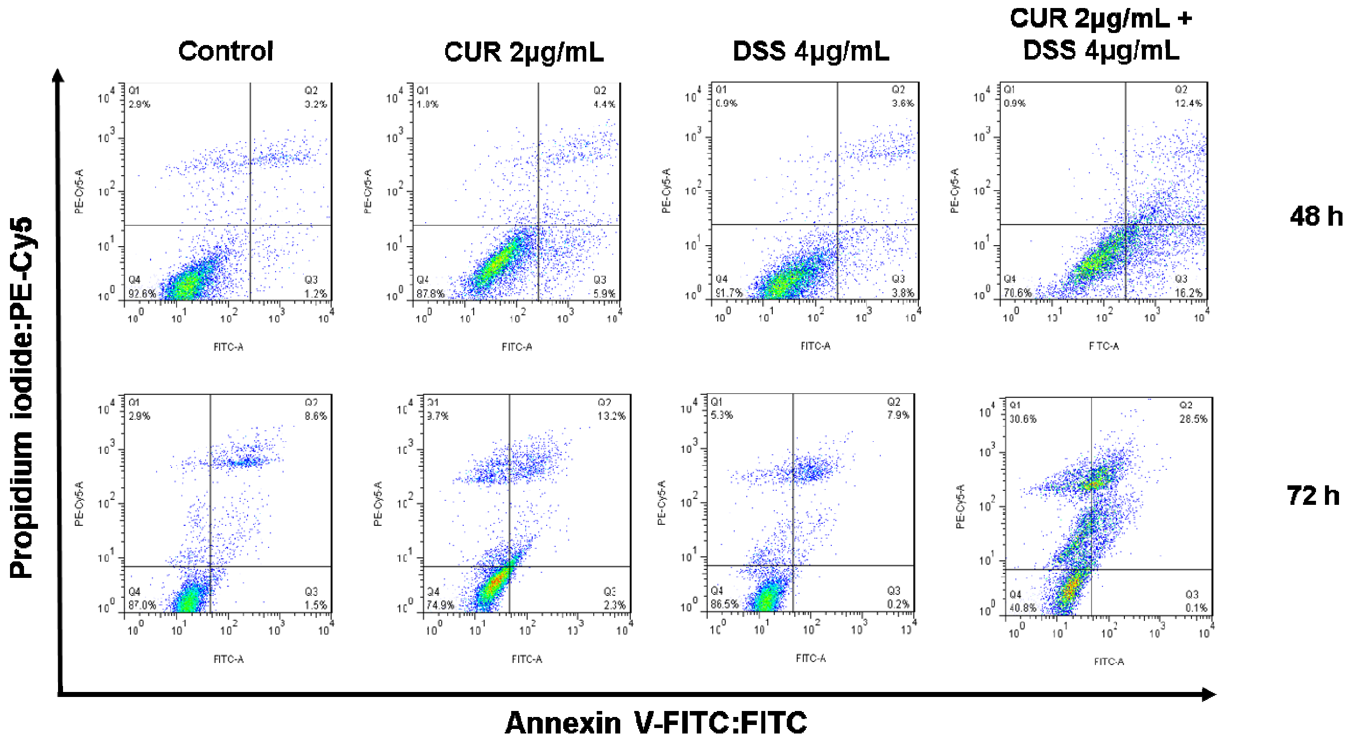


**Figure S7.** Representative dotplots of the effect of the combination of CUR with DSS at a ratio of 1:2 on the viability of B16-F10 cells measured by annexin V-FITC/PI staining. The control received 0.5% DMSO.

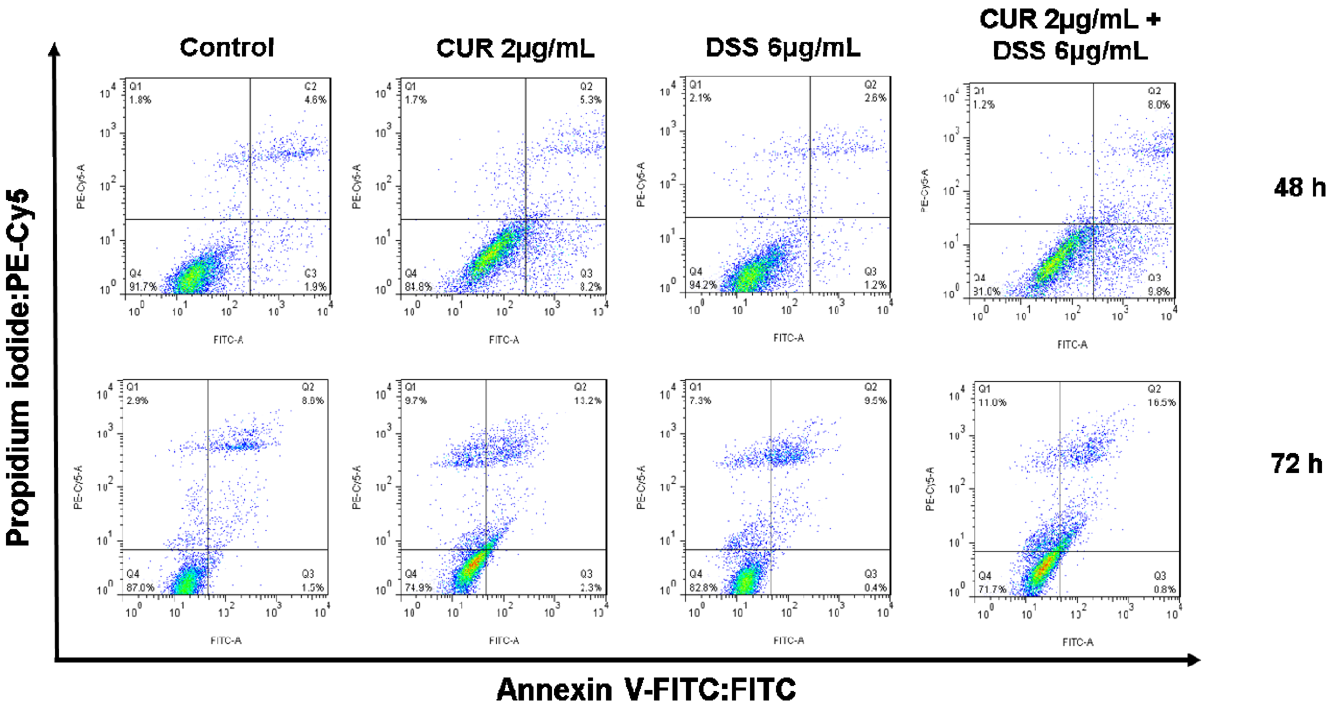


**Figure S8.** Representative dotplots of the effect of the combination of CUR with DSS at a ratio of 1:3 on the viability of B16-F10 cells measured by annexin V-FITC/PI staining. The control received 0.5% DMSO.

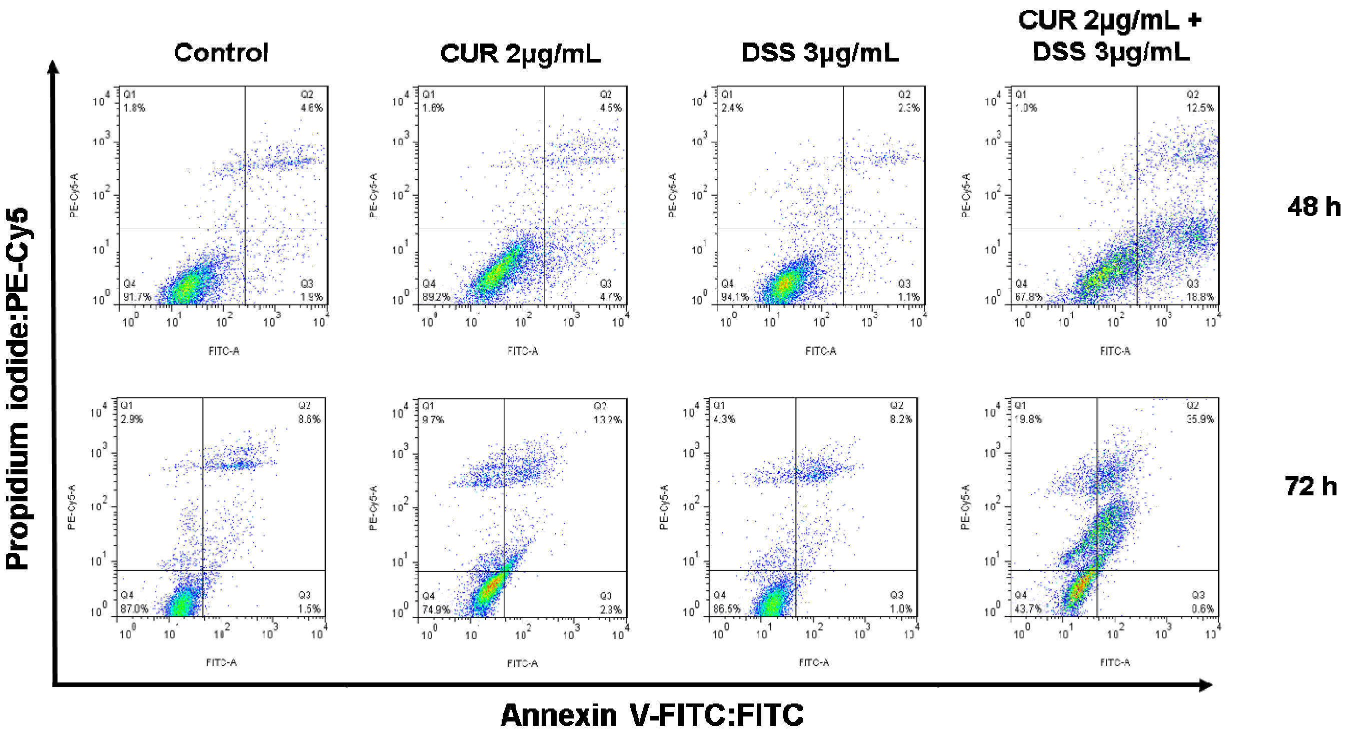


**Figure S9.** Representative dotplots of the effect of the combination of CUR with DSS at a ratio of 2:3 on the viability of B16-F10 cells measured by annexin V-FITC/PI staining. The control received 0.5% DMSO.

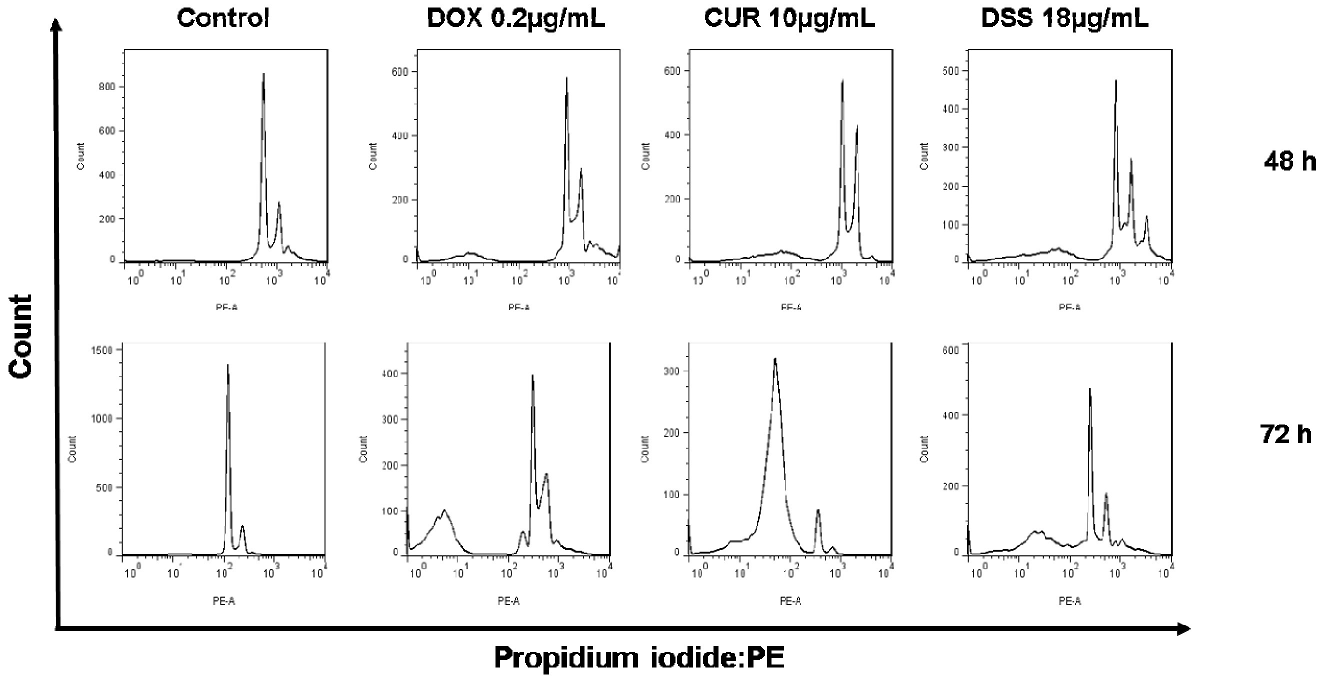


**Figure S10.** Representative histograms of the effect of CUR and DSS on DNA fragmentation and cell cycle distribution of B16-F10 cells. DOX was used as a positive control, and the negative control received 0.5% DMSO.

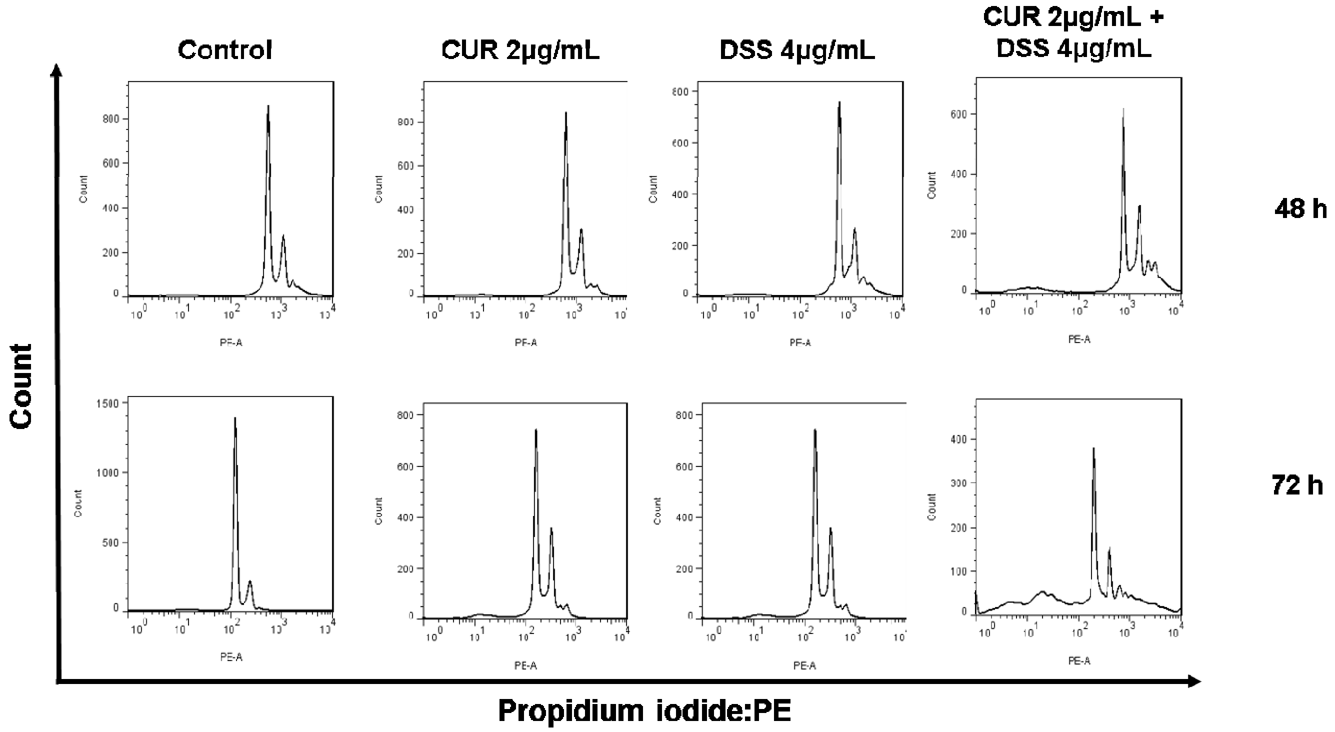


**Figure S11.** Representative histograms of the effect of the combination of CUR with DSS at a ratio of 1:2 on DNA fragmentation and cell cycle distribution of B16-F10 cells. The control received 0.5% DMSO.

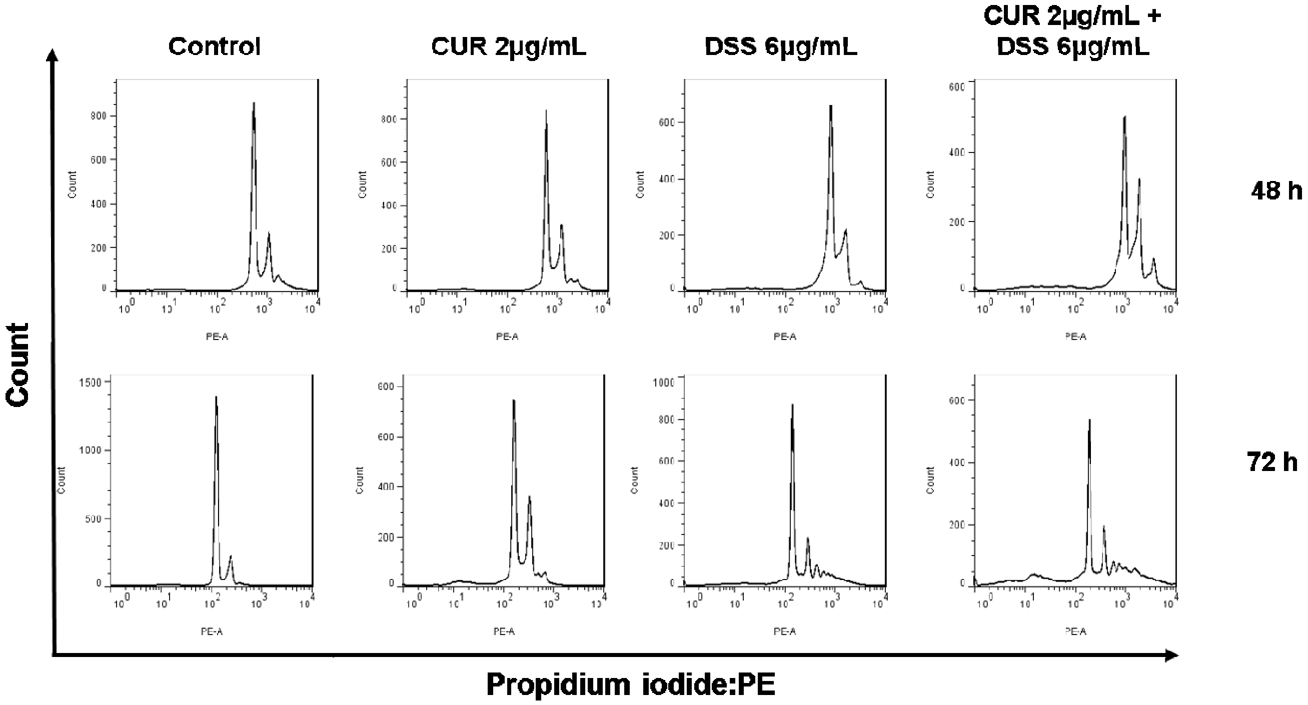


**Figure S12.** Representative histograms of the effect of the combination of CUR with DSS at a ratio of 1:3 on DNA fragmentation and cell cycle distribution of B16-F10 cells. The control received 0.5% DMSO.

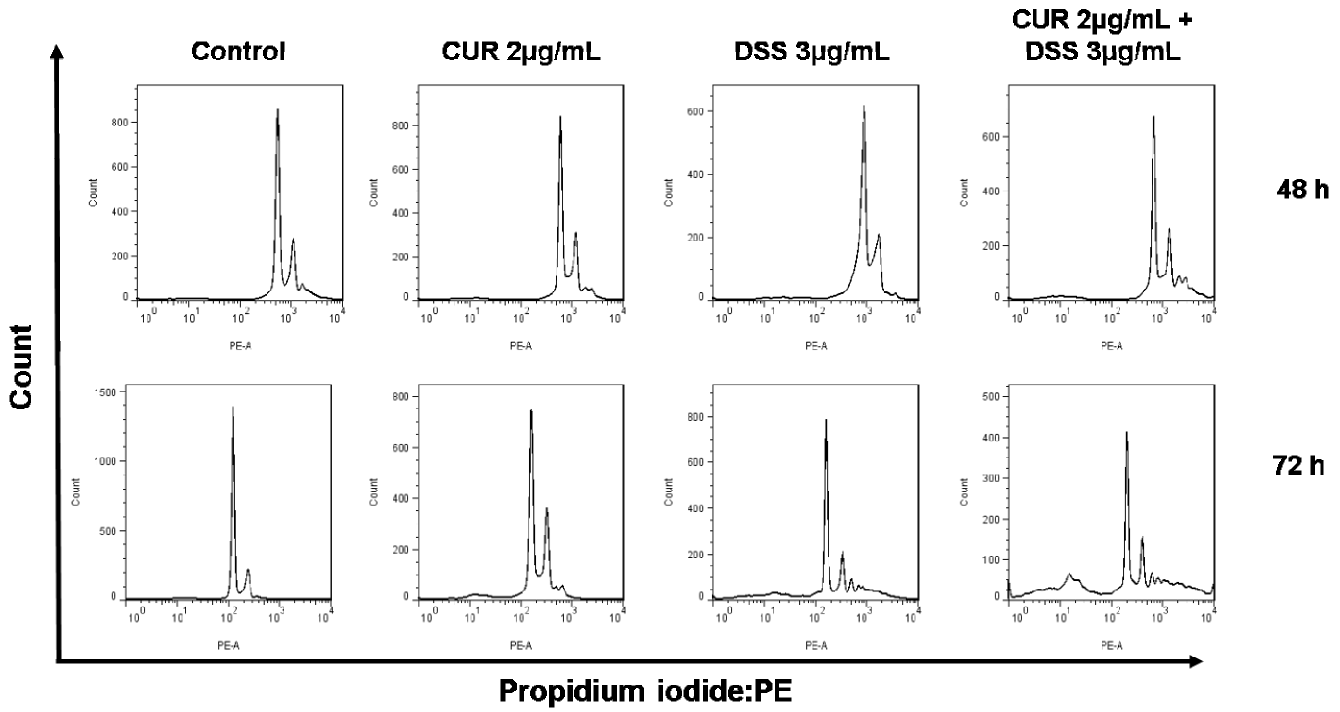


**Figure S13.** Representative histograms of the effect of the combination of CUR with DSS at a ratio of 2:3 on DNA fragmentation and cell cycle distribution of B16-F10 cells. The control received 0.5% DMSO.

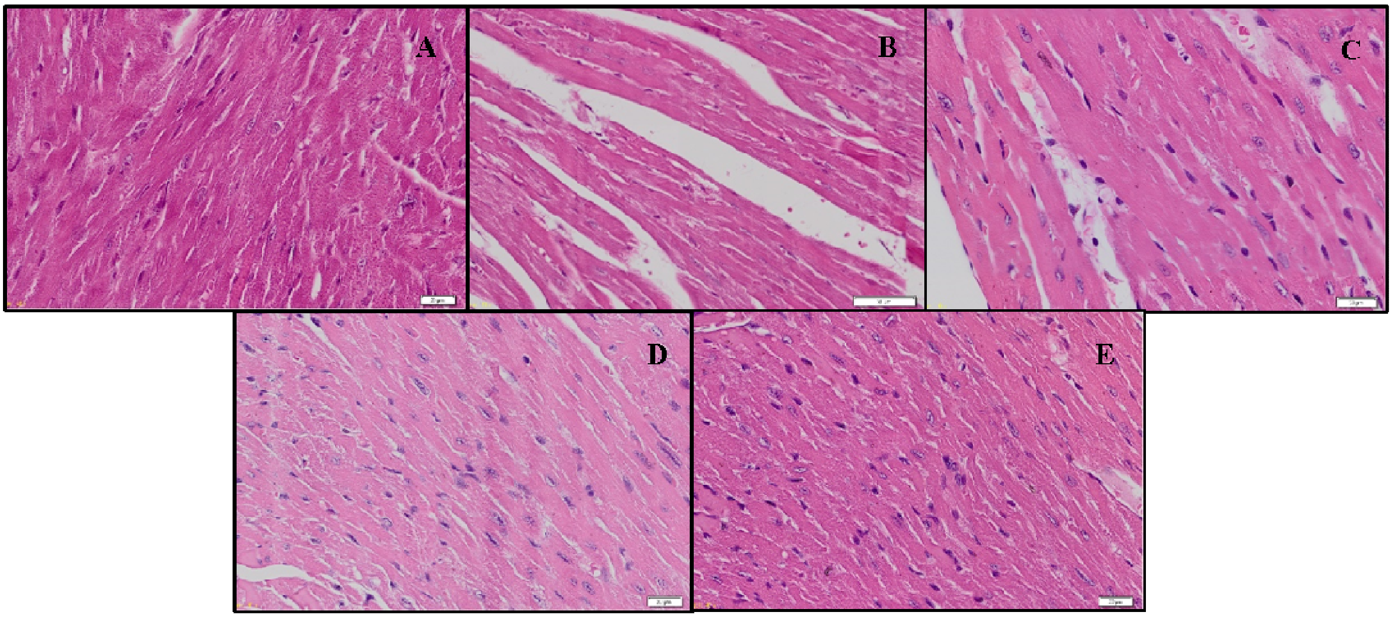


**Figure S14.** Representative histological analysis of hearts stained with hematoxylin and eosin and analyzed by light microscopy. The animals were treated with 5% DMSO (**A**), 1 mg/kg DOX (**B**), 20 mg/kg CUR (**C**), 60 mg/kg DSS (**D**) or 20 mg/kg CUR + 60 mg/kg DSS (**E**). Intact cardiomyocytes are observed with preservation of nuclei and transverse streaks observed longitudinally. Bars = 20 µm (**A**, **C**, **D** and **E**) or 50 µm (**B**).

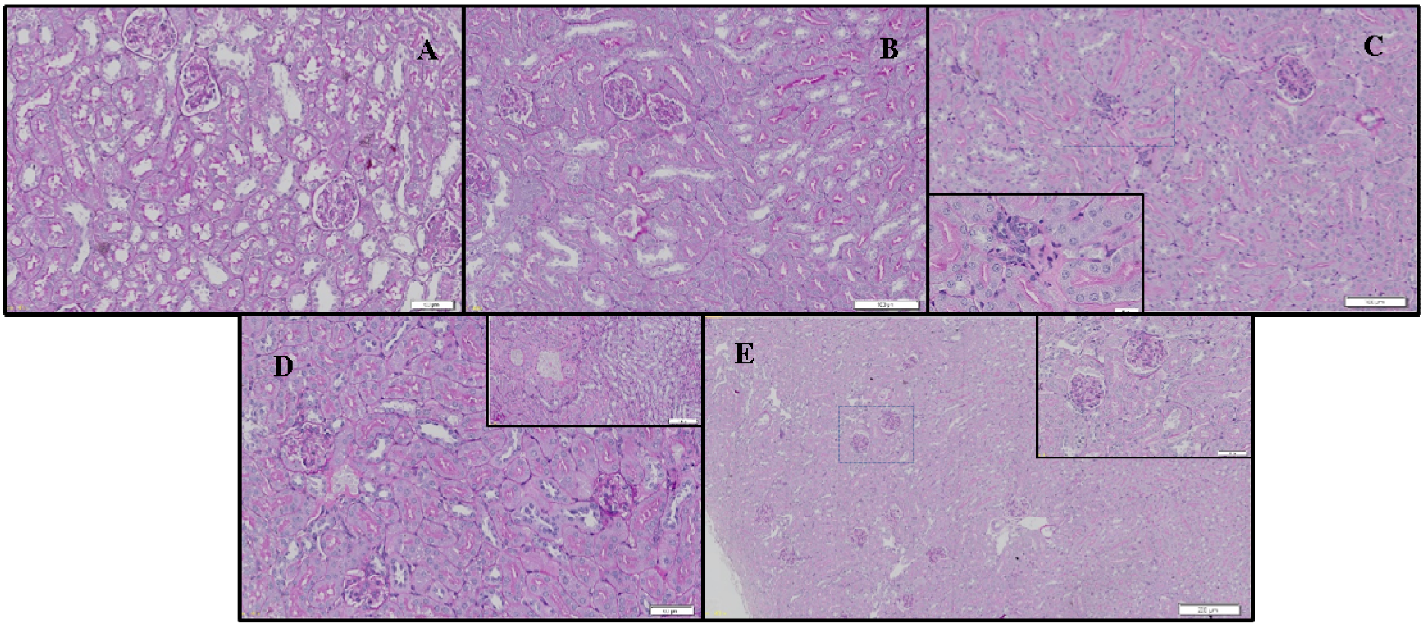

**Figure S15.** Representative histological analysis of kidneys stained with hematoxylin and eosin and analyzed by light microscopy. The animals were treated with 5% DMSO (**A**), 1 mg/kg DOX (**B**), 20 mg/kg CUR (**C**), 60 mg/kg DSS (**D**) or 20 mg/kg CUR + 60 mg/kg DSS (**E**). Bars = 50 µm (**A** and **D**), 100 µm (**B** and **C**) or 200 µm (**E**).

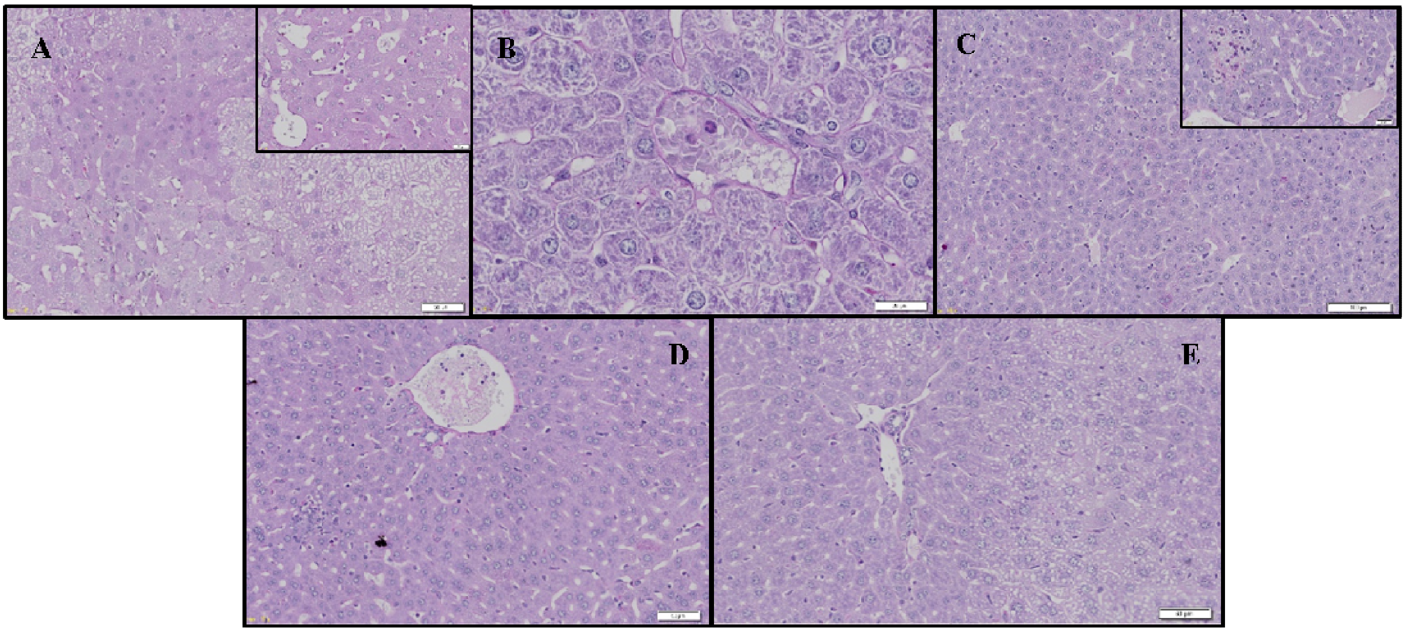


**Figure S16.** Representative histological analysis of livers stained with hematoxylin and eosin and analyzed by light microscopy. The animals were treated with 5% DMSO (**A**), 1 mg/kg DOX (**B**), 20 mg/kg CUR (**C**), 60 mg/kg DSS (**D**) or 20 mg/kg CUR + 60 mg/kg DSS (**E**). Bars = 50 µm (**A**, **D** and **E**), 20 µm (**B**) or 100 µm (**C**).

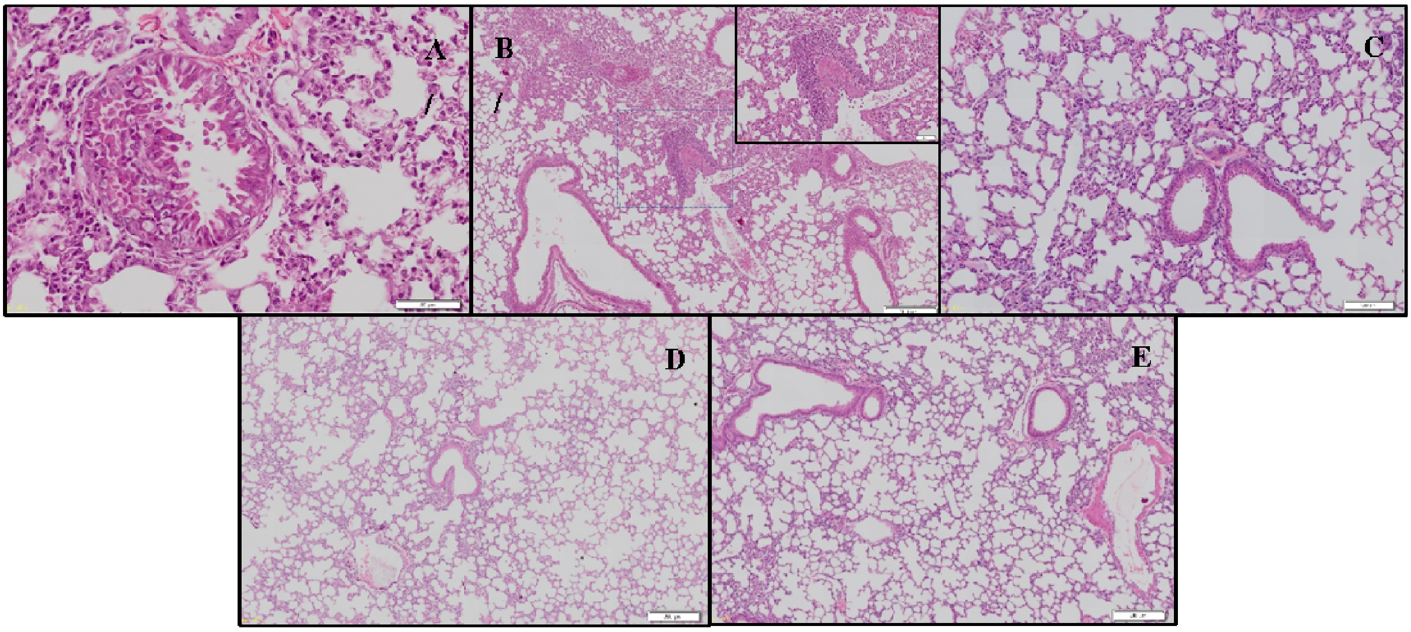


**Figure S17.** Representative histological analysis of lungs stained with hematoxylin and eosin and analyzed by light microscopy. The animals were treated with 5% DMSO (**A**), 1 mg/kg DOX (**B**), 20 mg/kg CUR (**C**), 60 mg/kg DSS (**D**) or 20 mg/kg CUR + 60 mg/kg DSS (**E**). Bars = 50 µm (**A**), 200 µm (**B**, **D** and **E**) or 100 µm (**C**).

**Table S1.** Inhibitory concentrations of CUR and DSS alone and in combination.

| | Inhibitory effect | CUR combinated (μg/mL) | CUR alone (μg/mL) | FIC of CUR | DSS combinated (μg/mL) | DSS alone (μg/mL) | FIC of DSS |
|---|---|---|---|---|---|---|---|
| Ratio 1:2 | 25% | 8.11 | 7.69 | 1.05 | 16.49 | 14.80 | 1.11 |
| | 50% | 1.79 | 9.69 | 0.18 | 3.98 | 16.49 | 0.24 |
| | 75% | 16.21 | 13.70 | 1.18 | 32.28 | 21.47 | 1.50 |
| Ratio 1:3 | 25% | 6.47 | 7.69 | 0.84 | 19.43 | 14.80 | 1.31 |
| | 50% | 2.76 | 9.69 | 0.28 | 8.30 | 16.49 | 0.50 |
| | 75% | 8.62 | 13.70 | 0.63 | 25.87 | 21.47 | 1.20 |
| Ratio 1:4 | 25% | 4.35 | 7.69 | 0.57 | 17.43 | 14.80 | 1.18 |
| | 50% | 3.74 | 9.69 | 0.39 | 15.00 | 16.49 | 0.91 |
| | 75% | 7.09 | 13.70 | 0.52 | 28.38 | 21.47 | 1.32 |
| Ratio 2:3 | 25% | 8.34 | 7.69 | 1.08 | 11.61 | 14.80 | 0.78 |
| | 50% | 1.96 | 9.69 | 0.20 | 3.18 | 16.49 | 0.19 |
| | 75% | 13.67 | 13.70 | 1.00 | 20.63 | 21.47 | 0.96 |
| Ratio 1:10 | 25% | 1.57 | 7.69 | 0.20 | 15.80 | 14.80 | 1.07 |
| | 50% | 2.08 | 9.69 | 0.21 | 20.83 | 16.49 | 1.26 |
| | 75% | 2.46 | 13.70 | 0.18 | 24.71 | 21.47 | 1.15 |

**Table S2.** Effect of CUR and DSS and their combination on body and relative organ weight from C57BL/6 mice bearing B16-F10 cells.

| | Control | DOX | CUR | DSS | CUR + DSS |
|---|---|---|---|---|---|
| Dose (mg/kg) | - | 1 | 20 | 60 | 20 + 60 |
| Survival | 10/10 | 09/10 | 09/10 | 09/10 | 07/10 |
| Initial body weight (g) | 21.60 ± 1.2 | 27.83 ± 0.4 | 25.65 ± 0.8 | 26.05 ± 0.6 | 23.23 ± 1.1 |
| Final body weight (g) | 26.83 ± 1.8 | 31.89 ± 0.9 | 29.89 ± 0.9 | 28.56 ± 0.8 | 26.71 ± 2.2 |
| Heart (g/100 g body weight) | 0.56 ± 0.05 | 0.39 ± 0.02 | 0.40 ± 0.01 | 0.69 ± 0.31 | 0.40 ± 0.05 |
| Liver (g/100 g body weight) | 5.42 ± 0.6 | 4.14 ± 0.2 | 4.06 ± 0.1 | 3.88 ± 0.2 | 4.21 ± 0.8 |
| Lung (g/100 g body weight) | 0.68 ± 0.07 | 0.51 ± 0.02 | 0.56 ± 0.06 | 0.57 ± 0.02 | 0.56 ± 0.07 |
| Kidneys (g/100 g body weight) | 1.27 ± 0.1 | 0.92 ± 0.04 | 1.01 ± 0.06 | 1.01 ± 0.04 | 1.03 ± 0.2 |

DOX was used as a positive control, and the negative control received 5% DMSO. Data are shown as the mean ± S.E.M. of 7-10 animals.

**Table S3.** Effect of CUR and DSS and their combination on hematological parameters of peripheral blood from C57BL/6 mice bearing B16-F10 cells

| | Control | DOX | CUR | DSS | CUR + DSS |
|---|---|---|---|---|---|
| Dose (mg/kg) | - | 1 | 20 | 60 | 20 + 60 |
| Erythrocytes ($10^6/mm^3$) | 5.8 ± 0.7 | 8.5 ±0.4* | 7.0 ± 0.7 | 9.0 ± 0.3** | 6.7 ± 0.6 |
| Hemoglobin (g/dL) | 10.2 ± 1.2 | 14.4 ± 0.9* | 12.4 ± 1.0 | 14.8 ± 0.4* | 12.6 ± 0.9 |
| Hematocrit (%) | 35.4 ± 4.5 | 47.7 ± 2.9 | 43.7 ± 4.2 | 49.6 ± 1.1 | 36.6 ± 6.5 |
| MCV (fL) | 63.8 ± 1.6 | 54.3 ± 1.5* | 61.9 ± 2.4 | 59.9 ± 1.5 | 60.6 ± 1.7 |
| Platelets ($10^3/mm^3$) | 55 ± 13 | 287 ± 77* | 83,3 ± 14 | 120 ± 40 | 188 ± 88 |
| Leukocytes ($10^3/mm^3$) | 3.5 ± 0.6 | 3.1 ± 0.5 | 2.6 ± 0.4 | 5.5 ± 0.6 | 4.6 ± 0.8 |
| Contagem diferencial de leucócitos (%) | | | | | |
| Lymphocytes | 53.4 ± 5.7 | 48.9 ± 3.6 | 63.5 ± 5.7 | 47.5 ± 4.9 | 51.4 ± 3.3 |
| Monocytes | 28.2 ± 2.2 | 24.8 ± 2.8 | 22.0 ± 2.9 | 34.2 ± 2.3 | 32.7 ± 2.1 |
| Granulocytes | 18.4 ± 5.3 | 26.3 ± 2.1 | 26.7 ± 12.5 | 18.2 ± 3.4 | 16.0 ± 3.3 |

DOX was used as a positive control. Data are shown as the mean ± S.E.M. of 7-10 animals. *$p < 0.05$ compared with control (5% DMSO) by ANOVA followed by Student Newman–Keuls test. MCV: Mean corpuscular volume.
